# Multi-omics Approaches to Deciphering a Hypervirulent Strain of *Campylobacter jejuni*

**DOI:** 10.1093/gbe/evt172

**Published:** 2013-11-06

**Authors:** Zuowei Wu, Orhan Sahin, Zhangqi Shen, Peng Liu, William G. Miller, Qijing Zhang

**Affiliations:** ^1^Department of Veterinary Microbiology and Preventive Medicine, Iowa State University; ^2^Department of Statistics, Iowa State University; ^3^U.S. Department of Agriculture, Agricultural Research Service, Western Regional Research Center, Albany, CA

**Keywords:** *Campylobacter jejuni*, comparative genomics, functional genomics, virulence evolution

## Abstract

*Campylobacter jejuni* clone SA recently emerged as the predominant cause of sheep abortion in the United States and is also associated with foodborne gastroenteritis in humans. A distinct phenotype of this clone is its ability to induce bacteremia and abortion. To facilitate understanding the pathogenesis of this hypervirulent clone, we analyzed a clinical isolate (IA3902) of clone SA using multi-omics approaches. The genome of IA3902 contains a circular chromosome of 1,635,045 bp and a circular plasmid of 37,174 bp. Comparative genomic analysis revealed that IA3902 is most closely related to *C. jejuni* NCTC11168, which is a reference strain and was previously shown to be non-abortifacient in pregnant animals. Despite the high genomic synteny and sequence homology, there are 12 variable regions (VRs) and 8,696 single-nucleotide polymorphisms and indels between the two genomes. Notably, the variable genes in the capsular polysaccharides biosynthesis and O-linked glycosylation loci of IA3902 are highly homogenous to their counterparts in *C. jejuni* subsp. *doylei* and *C. jejuni* G1, which are known to be frequently associated with bacteremia. Transcriptomic and proteomic profiles were conducted to compare IA3902 with NCTC11168, which revealed that the pathways of energy generation, motility, and serine utilization were significantly up-regulated in IA3902, whereas the pathways of iron uptake and proline, glutamate, aspartate, and lactate utilization were significantly down-regulated. These results suggest that *C. jejuni* clone SA has evolved distinct genomic content and gene expression patterns that modulate surface polysacharide structures, motilitiy, and metabolic pathways. These changes may have contributed to its hyper-virulence in abortion induction.

## Introduction

*Campylobacter jejuni* is a leading cause of bacterial foodborne gastroenteritis worldwide (Tauxe 1997; Adak et al. 2005). *Campylobacter* infections in humans are characterized by self-limiting watery or bloody diarrhea, abdominal cramps, nausea, and fever; whereas severe neurological sequelae, bacteremia, and other extraintestinal complications may develop infrequently (Peterson 1994; Nachamkin et al. 1998). The transmission of *C. jejuni* to humans is mainly via contaminated food of animal origin (Ogden et al. 2009). In addition, *Campylobacter* is also a cause of sheep abortion around the globe (Kirkbride 1993; Skirrow 1994). Historically, multiple *Campylobacter* species (primarily *C. fetus* subsp. *fetus*) and strains were associated with ovine abortion (Kirkbride 1993; Skirrow 1994); however, the proportion of *C. jejuni* isolates associated with sheep abortion has steadily increased since the late 1980s and outnumbered *C. fetus* subsp*. fetus* isolates in the United States at the end of last century (Kirkbride 1993; Delong et al. 1996). More importantly, our recent study indicated that a *C. jejuni* clone (SA for Sheep Abortion) has emerged as the predominant cause of *Campylobacter*-associated sheep abortion in the United States (Sahin et al. 2008), indicating that this clone is ecologically well adapted and hypervirulent in the ruminant host. Furthermore, zoonotic transmission of this emergent clone to humans via raw milk and other unknown routes has been recently demonstrated by molecular and epidemiological evidence, indicating that it is also a significant threat to public health (Sahin et al. 2012). Using a pregnant guinea pig model, the hyper-virulence of clone SA in inducing abortion was further demonstrated (Burrough et al. 2009), showing the extensive spread of clone SA beyond the gastrointestinal tract. These findings reveal a distinct phenotype of clone SA in causing systemic infection and are quite surprising as most *C. jejuni* strains are associated with gastrointestinal disease, instead of abortion.

During the past decade, genomic-based approaches have been commonly utilized to better understand the pathobiology of various pathogens including *C. jejuni*. Whole-genome sequencing studies revealed that *C. jejuni* genomes are in general syntenic and share a high degree of DNA sequence identity, but hypervariable regions and strain-specific genes and genomic islands are present (Parker et al. 2006). Furthermore, DNA microarray-based genomic comparisons showed that at least 21% of the total genes were highly divergent among *C. jejuni* strains (Dorrell et al. 2001; Parker et al. 2006). The strain-specific genes were mainly involved in the biosynthesis and modification of cell surface structures such as flagella, lipooligosaccharide (LOS), and capsular polysaccharide (CPS) (Dorrell et al. 2001; Gilbert et al. 2002; Karlyshev et al. 2005). How strain-specific genes and factors contribute to different disease presentations or enable *C. jejuni* to survive in unique ecological niches is still not well understood.

Functional genomics (whole-genome-based transcriptomics and proteomics) have also been utilized to complement comparative genomics to study various aspects of *Campylobacter* biology. Recent work indicated that the changes in global gene expression can alter the pathogenic potential of *C. jejuni* isolates and account for the significant phenotypic differences between genetically close *C. jejuni* isolates (Gaynor et al. 2004; Malik-Kale et al. 2007; Seal et al. 2007). Two variants of *C. jejuni* NCTC11168 were shown to be considerably different in virulence-associated phenotypes, including colonization, invasion, translocation, and motility, but no obvious genetic differences were found between them by multiple molecular genetics tools. However, transcriptional profiling of these two strains revealed significant differences in the expression pattern of genes involved in virulence (Gaynor et al. 2004). A similar situation was observed when the virulence and genetic properties were compared between a *C. jejuni* isolate recovered from a turkey and a *C. jejuni* isolate recovered from a chicken (Malik-Kale et al. 2007). Although these two *C. jejuni* isolates were indistinguishable by pulsed-field gel electrophoresis (PFGE), multi locus sequence typing (MLST), and microarray-based comparative genomic hybridization, significant differences in the expression profiles of genes were revealed by both microarray and two-dimensional differential in-gel electrophoresis (2D-DIGE) technology. These observations are in agreement with those obtained with other pathogenic bacteria, indicating that certain patterns of gene and/or protein expression are correlated with host specificity, adaptation to a specific niche, and pathogenic potential (Le Gall et al. 2005; Brinig et al. 2006).

Considering the distinct ability of *C. jejuni* clone SA to cause systemic infection and abortion, we hypothesize that this clone has evolved a unique genomic sequence (e.g., gene gain/loss, indel, and single-nucleotide polymorphisms [SNPs]) and functional features (e.g., gene and protein expression) as compared with non-abortifacient *C. jejuni* strains. To examine this hypothesis, we utilized a multifaceted holistic approach to profiling a clinical isolate (IA3902) of clone SA. From comparative genomic analysis, it was found that the genome of IA3902 was highly syntenic and homologous to that of NCTC11168, which was able to colonize the intestinal tract but unable to induce abortion (Burrough et al. 2009). Transcriptomic and proteomic analyses were further performed to compare IA3902 with phenotypically distinct NCTC11168. These findings provide important insights into the pathogenesis of *C. jejuni* clone SA.

## Materials and Methods

### Genome Sequencing, Gap Closure, Assembly, and Annotation

Genomic DNA of *C. jejuni* IA3902 was purified and shipped to 454 Life Sciences (Branford, CT), where the sequence was determined with a Genome Sequencer FLX instrument following the standard protocols (Margulies et al. 2005). The sequences obtained (single-end reads) were assembled de novo into unordered contigs by using the 454 Newbler Assembler software at the company. Blast analysis of the contigs against the NCBI-nr database indicated overall matches to known *C. jejuni* genome sequences. Each contig was locally aligned against the whole genome of *C. jejuni* NCTC11168 based on nucleotide homology, which resulted in tentative ordering of the contigs. Gaps between the contigs were closed by PCR amplification with Takara *Ex Taq* DNA polymerase (Clontech, Mountain View, CA) or *PfuUltra*HF DNA polymerase (Stratagene, La Jolla, CA) and subsequent Sanger sequencing in both directions. For closing certain gaps, direct sequencing using the genomic DNA as template was employed. The new sequences were assembled into the genome sequence using SeqMan from DNASTAR Lasergene (v.8; Madison, WI). Restriction enzyme digestion patterns obtained in silico with the newly assembled sequence were compared with those generated by pulsed field gel electrophoresis, to confirm the accuracy of the assembly. The J. Craig Venter Institute (JCVI, Rockville, MD) Annotation Service (http://www.jcvi.org/cms/research/projects/annotation-service/, last accessed November 19, 2013) was used for preliminary automatic assignment of function to genes. The automated annotation results were then manually inspected and curated using Manatee (http://manatee.sourceforge.net, last accessed November 19, 2013). The fully annotated complete genome and plasmid sequences were deposited at NCBI GenBank under the accession numbers CP001876.1 and CP001877.1.

### Phylogenetic Analysis and Comparative Genomics

Protein trees for genome-sequenced *C. jejuni* strains were generated from multiple alignments of the concatenated 12 conserved proteins (Brown et al. 2001; Santos and Ochman 2004), that is, initiation factor 2 (InfB); elongation factor G (FusA) and Tu (Tuf); ribosomal protein L2 (RplB), S5 (RpsE), S8 (RpsH), and S11 (RpsK); DNA topoisomerase I (TopA); signal recognition particle protein (Ffh); DNA gyrase B subunit (GyrB); GTP-binding protein LepA; and CTP synthase (PyrG) as described previously (Fouts et al. 2005). The phylogenetic tree was generated using PHYLIP version 3.69 (http://evolution.genetics.washington.edu/phylip.html, last accessed November 19, 2013) and was visualized by TREEVIEW (Page 1996). The whole-genome-based Blast tree was generated from the search using the genome of IA3902 against the database of complete *C. jejuni* genome in the NCBI BlastN web server (http://blast.ncbi.nlm.nih.gov, last accessed November 19, 2013). Protein homology searches were performed using BlastP (Camacho et al. 2009). The synteny comparison and SNP analyses were carried out using MUMmer (Kurtz et al. 2004).

### Microarray, Labeling, and Hybridization

*C. jejuni* version 3 DNA pan-genome slides were supplied by JCVI (http://pfgrc.jcvi.org, last accessed November 19, 2013). In total, 6,987 70-mer probes were included in the array, among which 2,088 were based on the open reading frames (ORFs) of NCTC11168 and the rest were based on the ORFs of *C. jejuni* RM1221, 81-176, CG8421 and *C. jejuni* subsp. *doylei* 269.97. Each probe was spotted in duplicates. Due to the differences (SNPs, indels, etc.) in nucleotide sequences between IA3902 and NCTC11168, 1,525 of the 2,088 probes of NCTC11168 perfectly matched to the genes of IA3902. RNA was extracted from *C. jejuni* cells incubated for 20 h on Mueller-Hinton (MH) agar. cDNA synthesis and labeling, hybridization, and washing were performed following the standard protocol from JCVI. A dye swap strategy was employed in alternating replicate slides.

### Data Extraction and Analysis

Microarray slides were scanned at 532-nm (Cy3) and 635-nm (Cy5) wavelengths using a ScanArray3000 scanner (PerkinElmer, Waltham, MA) at 10 µm resolution. Fluorescence intensities of each spot were extracted using the Imagene 8.0 software (Axon Instruments, Foster City, CA). Analysis of the microarray data was conducted as follows: 1) After background subtraction, all spots with fluorescent median intensities which were lower than the background median plus two times the standard deviation in both channels were removed from the final data analysis. 2) The fluorescence intensity in each wavelength was log2 transformed and normalized using locally weighted linear regression (lowess) performed using statistical software R (http://www.r-project.org, last accessed November 19, 2013). 3) For genes with more than one probe, only the genes with all the probes showing the same regulation trend were kept for further analysis. 4) Moderated *t*-test was performed using the limma package in R to identify the differentially expressed genes (Smyth 2004). In this study, fold change >2 and *q* value <0.1 (FDR controlled at 10%) were used as the cutoff criteria for differentially expressed genes. Data were submitted to the GEO database (accession number GSE45601).

### Real-Time Quantitative Reverse Transcriptase-Polymerase Chain Reaction

Microarray expression data for a subset of genes were verified by real-time quantitative reverse transcriptase-polymerase chain reaction (qRT-PCR) using the iQ SYBR Green Super Mix (Bio-Rad, Hercules, CA) and MyiQTM instrument (Bio-Rad). Oligonucleotide primers were designed using Primer3 software (Rozen and Skaletsky 2000). Primer pairs which target the conserved region of a gene in both IA3902 and NCTC11168 were selected. Relative expression of each target gene was normalized to expression of the 16S rRNA gene. Amplification efficiency and relative transcript abundance were calculated as previously described (Livak and Schmittgen 2001).

### 2D-DIGE and Mass Spectroscopy

2D DIGE was performed to compare the expression of whole-cell proteins of *C. jejuni* strains IA3902, IA5908, and NCTC11168 at Applied Biomics Inc. (Hayward, CA) following the established methods (Siboo et al. 2008; Banerjee et al. 2012). Briefly, 20 h cultures of bacteria were harvested from MH plates and centrifuged. The cell pellets were washed with phosphate buffered saline four times and then were dissolved in 2D lysis buffer (7 M urea, 2 M thiourea, 4% CHAPS, and 30 mM Tris–HCl, pH 8.5). Total proteins of each strain were extracted from the lysed cells and equal amount of protein extracts from each strain were labeled with Cy2, Cy3, or Cy5 (GE Healthcare, Piscataway, NJ), respectively. The labeled protein extracts were mixed and subjected to isoelectric focusing on a 13-cm precast nonlinear IPG (immobilized pH gradient) strip (pH 3–10, Amersham) and then were separated on gradient sodium dodecyl sulfate (SDS) gel (the 9–12% SDS gel was prepared using low-florescent glass plates). After electrophoresis, the gels were scanned immediately using Typhoon Trio scanner (Amersham Biosciences), and images were analyzed using ImageQuant and DeCyder software version 6.0 (GE-Healthcare). The ratio change of the protein differential expression was obtained from in-gel DeCyder software analysis. Protein spots of interest (with the threshold of 1.5-fold change in fluoresce intensity between strains) were excised from the gel using an Ettan spot picker (Amersham Biosciences) and then were subjected to in-gel trypsin digestion, peptides extraction, and desalting, followed by mass spectrometry (MALDI-TOF/MS-MS). Peptides were analyzed with the MASCOT and NCBI-nr database to determine the identity of the corresponding protein for each spot.

### Immunoblotting Analysis of CfrA

*C. jejuni* NCTC11168 and four isolates of clone SA (IA3902, IDB099A, VDL768, and SD4183) were cultured in MH broth or in MH broth supplemented with an iron chelator (20 µM desferrioxamine). Cells were collected and resuspended in SDS-PAGE loading buffer. The expression levels of CfrA were analyzed by Western blotting using anti-CfrA and anti-MOMP antibodies as described previously (Zeng et al. 2009).

## Results

### Genomic Features of *C. jejuni* IA3902

*C. jejuni* IA3902 (a prototypical isolate of clone SA) is a fresh clinical isolate obtained from an aborted sheep placenta and was shown to be highly virulent in inducing abortion in a pregnant guinea pig model (Sahin et al. 2008; Burrough et al. 2009). Two rounds of sequencing on a Genome Sequencer FLX machine generated an average 55-fold coverage depth for the genome (supplementary table S1, Supplementary Material online). The sequences were assembled into 37 contigs. Except for eight contigs, which were largely the small ones that showed some degree of homology to capsular loci in various *Campylobacter* species, all other contigs were tentatively ordered using the *C. jejuni* NCTC11168 genome as a scaffold. All of the gaps between the ordered contigs were closed by Sanger sequencing of PCR products and/or direct sequencing on genomic DNA. The gap corresponding to the capsular region was successfully closed by a series of PCR and conventional sequencing. The assembled genome is composed of a circular chromosome of 1,635,045 bp and a circular plasmid of 37,174 bp (supplementary table S2, Supplementary Material online). The in silico enzyme digestion patterns (with *KpnI* and *SmaI*) of the genome sequence were virtually identical to those generated by pulsed field gel electrophoresis (data not shown), validating the final genome assembly. Annotation of the genome (automated annotation followed by manual curating) indicated the presence of 1,613 chromosomal and 53 plasmid putative coding sequences. As with other *C. jejuni* strains, IA3902 chromosomal genome has a relatively low GC content (30.57%) and a dense genome (93% of it codes for proteins). Additionally, it contains 44 tRNAs, 3 rRNA operons, and 4 miscellaneous RNAs (supplementary table S2, Supplementary Material online).

### Phylogenetic Analyses and Comparative Genomics

**Fig. 1.—:**
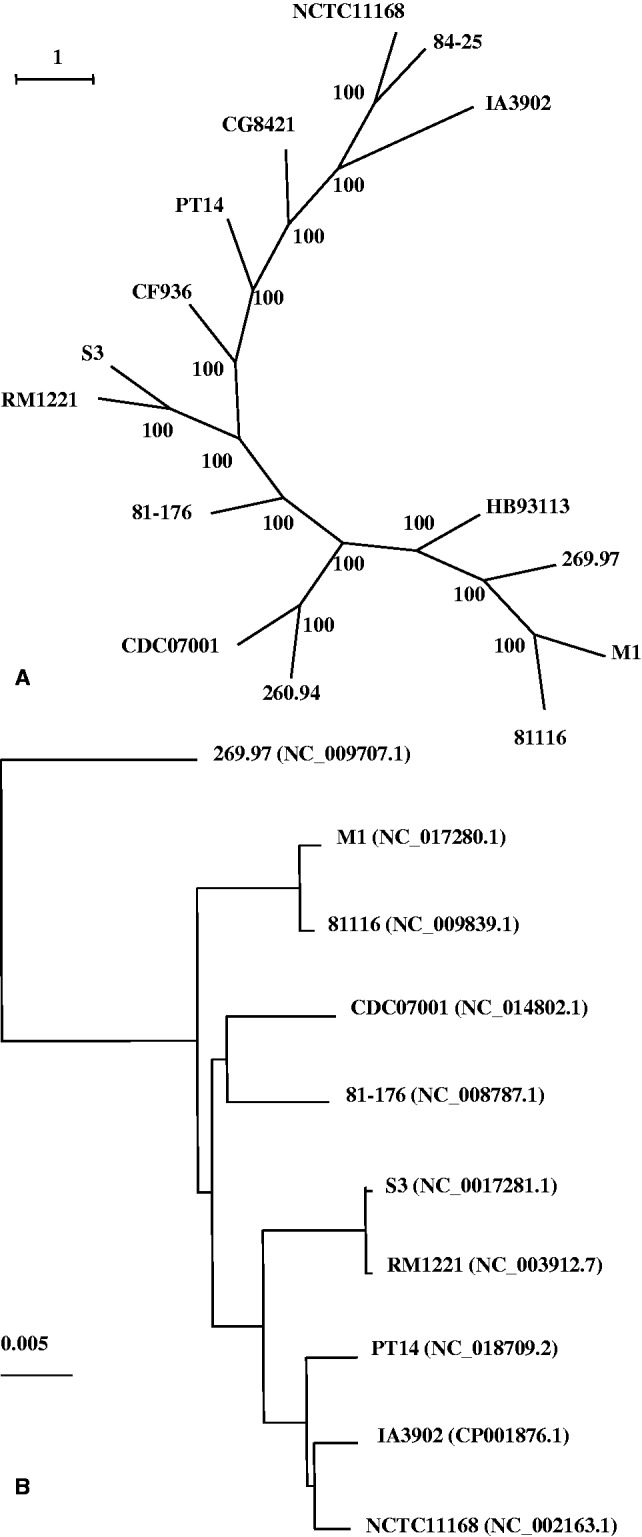
Phylogenetic analysis of *Campylobacter jejuni* IA3902 and other genome-sequenced strains. (*A*) The maximum-likelihood protein tree for genome-sequenced *C. jejuni* strains were generated from concatenated multiple alignments of 12 conserved proteins (InfB, FusA, Tuf, RplB, RpsE, RpsH, RpsK,TopA, Ffh, GyrB, LepA, and PyrG). The numbers along the branches of the tree denote percent occurrence of nodes among 100 bootstrap replicates. The scale bar represents the number of amino acid substitutions per site. (*B*) A dendrogram generated from whole genomic Blast shows the genetic relationship of *C. jejuni* strains, for which whole-genome sequences are available in GenBank. Genome accession numbers are shown in the parentheses. The scale bar represents the number of nucleotide substitutions per site.

To analyze the phylogenic relationship between IA3902 and the other genome-sequenced *C. jejuni* strains, we constructed a maximum-likelihood concatenated protein tree using a set of 12 conserved protein sequences that have been previously shown to be reliable markers for phylogenetic analysis (Brown et al. 2001; Santos and Ochman 2004; Fouts et al. 2005). The results showed that IA3902 is closely related to *C. jejuni* strains NCTC11168 and 84-25 (fig. 1*A*). NCTC11168 was originally isolated from a diarrheic patient and was previously demonstrated to be non-abortifacient in pregnant animals (Burrough et al. 2009), and 84-25 was isolated from human cerebrospinal fluid (http://www.ncbi.nlm.nih.gov/genome, last accessed November 19, 2013). Whole-genome-based Blast analysis using the completely sequenced genomes of *C. jejuni* available at NCBI also showed that IA3902 is most closely related to NCTC11168 (fig. 1*B*).

**Fig. 2.—:**
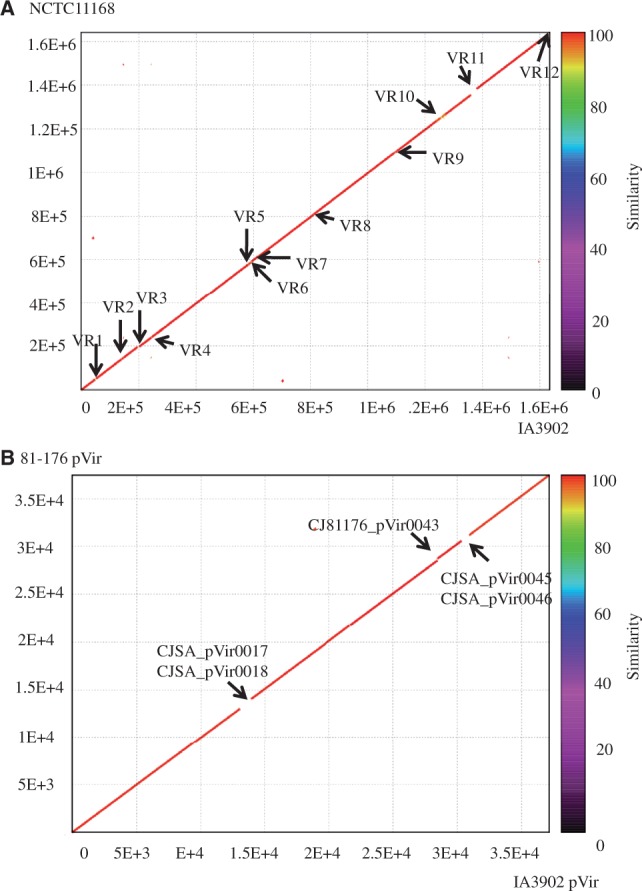
Whole genome comparison between *Campylobacter jejuni* IA3902 and *C. jejuni* NCTC11168 (*A*) and the comparison between *pVir* of *C. jejuni* IA3902 and *pVir* of *C. jejuni* 81-176 (*B*). Strain-specific regions (VRs) are indicated by discontinuities on the diagonal line (marked by arrows). The numbers along the axes denote the genomic coordinates of the strains.

The chromosome of *C. jejuni* IA3902 is highly syntenic and similar to that of *C. jejuni* NCTC11168, without any noticeable genomic translocation or inversion (fig 2*A*). The plasmid of *C. jejuni* IA3902 is highly homologous to the *pVir* plasmid of *C. jejuni* 81-176, with only five different genes (fig 2*B* and supplementary table S3, Supplementary Material online). The striking level of homology between the IA3902 and NCTC11168 genomes even includes the genes within the LOS biosynthesis cluster which is commonly variable between *C. jejuni* strains (Parker et al. 2005). However, a close examination of the two genomes revealed 12 small VRs, involving 72 genes, between these two strains (fig 2A and supplementary table S3 and fig. S1, Supplementary Material online). Of the variable genes, 25 genes are specific for IA3902, 32 genes are specific for NCTC11168, and 15 have homologs in both strains, but their sequences are significantly different (more than 15% divergence in aa sequence). Most of the strain-specific genes are located in the flagellin/motility locus (VR10) and the CPS locus (VR11) (supplementary table S3, Supplementary Material online). Of note, presence of *fspA* gene varies among *C. jejuni* strains, and the encoded protein is secreted through the flagellar system (Poly et al. 2007). However, only the FspA2 variant, present in IA3902 (CJSA_0814), has been shown to be associated with apoptosis (Poly et al. 2007). NCTC11168 has the FspA1 (Cj0859c) variant.

Another interesting and relatively unique feature of IA3902 is a chromosomal insertion that contains the tetracycline resistant gene (*tetO*, *cjsa_0193*) in the chromosome. The *tetO* gene and the adjacent region that putatively encodes IS*605* family transposases and TnpV were inserted into the 5′ coding region of *cjsa_0191* (corresponding to *cj0203c* in NCTC11168) of the IA3902 genome. Although a chromosomal location of *tetO* has been occasionally reported in *Campylobacter*, it is usually located on conjugative plasmids (e.g., *pTet*) (Gibreel et al. 2004; Poly et al. 2008). Further PCR analysis confirmed the invariable insertion of *tetO* into the same chromosomal locus (*cjsa_0191*) in all clone SA isolates collected since 2003 (over 100 isolates), all of which are resistant to tetracyclines (minimum inhibitory concentration [MIC] ≥16 µg/ml).

Additionally, due to the presence of small insertions/deletions (indels) or mutations, seven genes were annotated as pseudogenes in NCTC11168, but these are predicted to be functional in IA3902; conversely, 12 genes were annotated as pseudogenes in IA3902 but not in NCTC11168 (supplementary table S3, Supplementary Material online). Majority of these genes were predicted to encode putative or hypothetical proteins, except for *cjsa_1007* (nitroreductase family protein) and *arsR* in IA3902 and *cj1110c* (MCP-type signal transduction protein) in NCTC11168. Besides the above variations, 8696 SNPs or indels were identified between the homologous genes of IA3902 and NCTC1168. 2,605 SNPs were identified as nonsynonymous SNPs and distributed among 565 genes; 455 SNPs or indels were found to be located at the promoter regions of 128 genes (supplementary fig. S2, Supplementary Material online). The average nonsynonymous SNPs per kb for each gene are apparently higher in the genes associated with defense mechanisms, cell wall/membrane biogenesis, cell motility, inorganic ion transport and metabolism, and unknown function category (supplementary fig. S2, Supplementary Material online). Overall, these results showed that the genome of IA3902 is highly syntenic with the genome of NCTC11168 (fig. 2*A*) and that it does not harbor any known pathogenicity islands or virulence genes that are known to be associated with induction of abortion (Grogono-Thomas et al. 2003; van Putten et al. 2009), suggesting that IA3902 has evolved novel virulence mechanisms.

### Transcriptomic Comparisons between IA3902 and NCTC11168

**Fig. 3.—:**
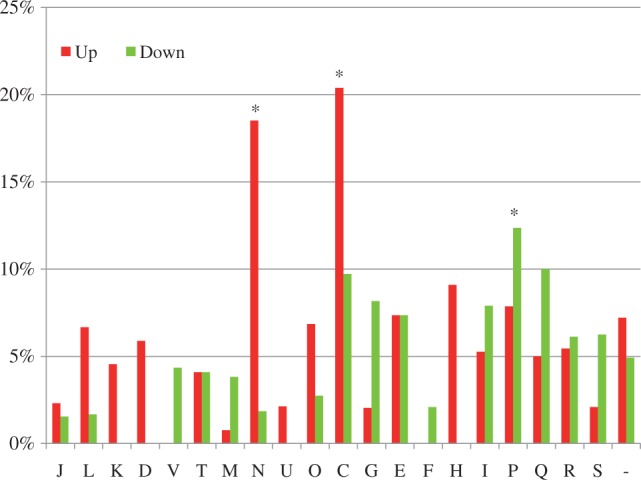
Gene enrichment analysis of the differentially expressed genes between *Campylobacter jejuni* IA3902 and NCTC11168 detected by microarray. COG category codes are indicated on the *x* axis. The fraction of genes in each COG category is shown on the *y* axis. Red bars indicate genes up-regulated in IA3902, and green bars indicate genes down-regulated in IA3902. COG categories that are significantly enriched (*P* < 0.05, Fisher’s exact test) are indicated by an asterisk. COG category codes: J, translation; L, replication, recombination, and repair; K, transcription; D, cell cycle control, mitosis, and meiosis; V, defense mechanisms; T, signal transduction mechanisms; M, cell wall/membrane biogenesis; N, cell motility; U, intracellular trafficking and secretion; O, posttranslational modification, protein turnover, chaperones; C, energy production and conversion; G, carbohydrate transport and metabolism; E, amino acid transport and metabolism; F, nucleotide transport and metabolism; H, coenzyme transport and metabolism; I, lipid transport and metabolism; P, inorganic ion transport and metabolism; Q, secondary metabolites biosynthesis, transport and catabolism; R, general function prediction only; S, function unknown; and -, not in COGs.

To explore the differences in global gene expression patterns between IA3902 and NCTC11168, their transcriptomes in culture media were compared using DNA microarray. In total, 108 genes were found to be up-regulated (supplementary table S4, Supplementary Material online) and 114 genes were found to be down-regulated in IA3902, when compared with NCTC11168. However, as the microarray probes were designed based on the NCTC11168 genome sequence, several IA3902 genes were represented by mismatch probes because of the nucleotide differences between IA3902 and NCTC11168. Although the up-regulated genes with mismatch probes are to represent the true expression changes (i.e., the true expression of the gene may be tempered by the mismatch) in these genes of IA3902, the down-regulated genes with mismatch probes are not necessarily identified accurately in the microarray, and therefore they were removed from the down-regulated gene list. This reduced the down-regulated gene number to 81 (supplementary table S4, Supplementary Material online). Clusters of Orthologous Groups (COG) functional classification and enrichment analysis of the differentially expressed genes revealed that up-regulated genes were statistically enriched in the “Cell motility” and “Energy production and conversion” functional categories while down-regulated genes were statistically enriched in the “Inorganic ion transport and metabolism” functional class (fig. 3). In order to confirm microarray data, 11 genes representing a large range of fold change in gene expression as determined by microarray were selected for qRT-PCR analysis (supplementary table S5, Supplementary Material online). High correlation was obtained between the microarray and the qRT-PCR data (*R*^2 ^= 0.8853), independently confirming the microarray results for the subset of genes tested (supplementary fig. S3, Supplementary Material online).

The largest group of genes with increased expression in IA3902 was related to the functional category “Energy production and conversion.” Many of these genes are associated with respiration. *nuoB*, *nuoC*, *nuoH*, *nuoJ*, and *nuoN* encode the components of the proton pump Complex I (also termed NADH:ubiquinone oxidoreductase) of respiratory chains, which is known to pass the electrons from α-ketoglutarate respiration to the electron transport chain (Weingarten et al. 2008). Hydrogenase subunits encoding genes (*hydA*, *hydB*, and *hydC*), formate dehydrogenase subunits encoding genes (*fdhA*, *fdhB*, and *fdhC*), and 2-oxoglutarate:acceptor oxidoreductase subunit encoding gene (*oorA*) are involved in the respiration with hydrogen, formate, and α-ketoglutarate as electron donors, respectively (Weingarten et al. 2008). *frdB* and *nrfH* encode reductases in anaerobic branched electron transport chains. *frdB* is involved in the respiration with fumarate as a terminal electron acceptor and *nrfH* is involved in the respiration with nitrate and nitrite as electron acceptors (Weingarten et al. 2008). Moreover, several proteins in this category related to oxidation–reduction reactions were also shown to be overexpressed in IA3902. *cjsa_0012* (*cj0012c*; *rrc*) encodes a protein which protects anaerobic microorganisms against oxidative stress (Yamasaki et al. 2004). *cjsa_1497* (*cj1586*; *cgb*) encodes a hemoglobin which oxidizes and detoxifies nitric oxide against nitrosative stress (Elvers et al. 2005). *fdxA* (encoding a [Fe–S] protein) is involved in the oxidative stress defense of *C. jejuni* (van Vliet et al. 2001), but the functions of *fdxB* (encoding a [Fe-S] protein) and *cjsa_0823* (*cj0874c*; encoding a putative cytochrome C) remain unknown. The up-regulated genes *iscS* and *iscU* are critical in [Fe–S] cluster biosynthesis of iron–sulfur proteins (Frazzon et al. 2002).

Another group of up-regulated genes in the abortion strain IA3902 is associated with the functional category “Cell motility,” including genes encoding the flagellar hook protein FlgE and FlgK; the flagellar basal body protein FlgH, FlgG, FlgG2, and FlgA; the flagellar capping protein FliD; the filament-associated protein FlaG; the flagellar export chaperone FliS (Lam et al. 2010); anti-σ^28^ factor FlgM; the energy taxis protein CetA (Elliott and Dirita 2008); and a FliA (σ^28^)-dependent gene encoding a hypothetical protein (CJSA_0364; Cj0391c) (Carrillo et al. 2004).

The major group of significantly down-regulated genes in IA3902 is the “Inorganic ion transport and metabolism” functional class. Besides three ABC transporter genes *cjsa_0132*, *cjsa_0134*, and *cjsa_0730* (corresponding to *cj0141c*, *cj0143c*, and *cj0774c* in NCTC11168, respectively), this group mainly included the iron transport genes. In *Campylobacter*, several iron-acquisition systems have been identified. Both IA3902 and NCTC11168 encode a Fe(II)-transport system (FeoAB), a hemin/hemoglobin-uptake system (ChuABCDZ), a ferric enterobactin uptake system (CeuBCDE and CfrA), a ferri-transferrin/lactoferrin uptake system (CJSA_0163-0168; Cj0173c–0178c), and a ferric-rhodotorulic acid uptake system (CJSA_1569-1574; Cj1658-1663) (Miller et al. 2009). In strain IA3902, except the Fe(II)-transport system, all other iron uptake systems are in the down-regulated genes, represented by *chuA*, *chuB*, *chuD*, and *chuZ*; *ceuD* and *cfrA*; *cfbpA*, *cfbpB* (*cjsa_0164-0165*; *cj0174c-cj0175c*), and *cjsa_0166* (*cj0176c*, a putative lipoprotein); and *cjsa_1570-1574* (*cj1659-1663*). Moreover, the genes of the energy-transduction systems of iron transport, *exbB3*, *exbB2*, and *exbD2*, were also down-regulated in IA3902.

The genomic analysis also showed that similar to NCTC11168, IA3902 possesses the genes encoding enzymes of the complete catabolism of amino acids (data not shown). Transcriptomic analysis suggested that significant metabolic differences exist between IA3902 and NCTC11168. In *C. jejuni*, the transmembrane transport and catabolism of l-serine to pyruvate and ammonia are carried out by a two-gene operon encoding serine transporter (SdaC) and serine dehydratase (SdaA) (Velayudhan et al. 2004). Proline is transported by a Na(+)/proline symporter (PutP) and oxidized to glutamate via a bifunctional proline dehydrogenase (PutA) (Guccione et al. 2008). The major route for aspartate and glutamate transport is the PEB1 system, which is encoded by a four-gene operon of *pebC*, *peb1A*, and two ABC-type amino-acid transporter permease genes (Leon-Kempis Mdel et al. 2006). Intracellular glutamate is then transaminated by aspartate aminotransferase AspB into aspartate and aspartate is catalyzed by aspartase AspA into fumarate (Guccione et al. 2008). *C. jejuni* also contains the C4-dicarboxylate antiporter systems, DcuA and DcuB, which are necessary for fumarate:succinate antiport during fumarate respiration under oxygen-limited conditions (Guccione et al. 2008). Compared with NCTC11168, IA3902 up-regulated the serine utilization genes *sdaC* and *sdaA* and C4-dicarboxylate antiporter gene *dcuB* but down-regulated proline, aspartate, and glutamate utilization genes, *putP* and *putA*, the *peb1* system (*cjsa_0864-0867*; *cj0919c-0922c*), and *aspB*. Differences between the two strains are also present in the metabolism of organic acid, l-lactate. In *C. jejuni* NCTC11168, l-lactate is transported primarily via the LctP permease and the intracellular oxidation of l-lactate to pyruvate is catalyzed by the action of both an iron–sulfur protein complex (*cj0075c-73c*) and an iron–sulfur containing membrane-associated oxidoreductase (*cj1585c*) (Thomas et al. 2011). In IA3902, *lctP* and iron–sulfur protein complex subunits encoding genes (*cjsa_0067-0068*; *cj0074c-0075c* in NCTC11168) were shown to be down-regulated, suggesting down-regulation of lactate metabolism. Moreover, genes encoding aconitate hydratase (CJSA_0790, AcnB) and fumarate hydratase (CJSA_1298, FumC) in the citric acid cycle were down-regulated in IA3902.

### Proteomic Comparisons between IA3902 and NCTC11168

**Fig. 4.—:**
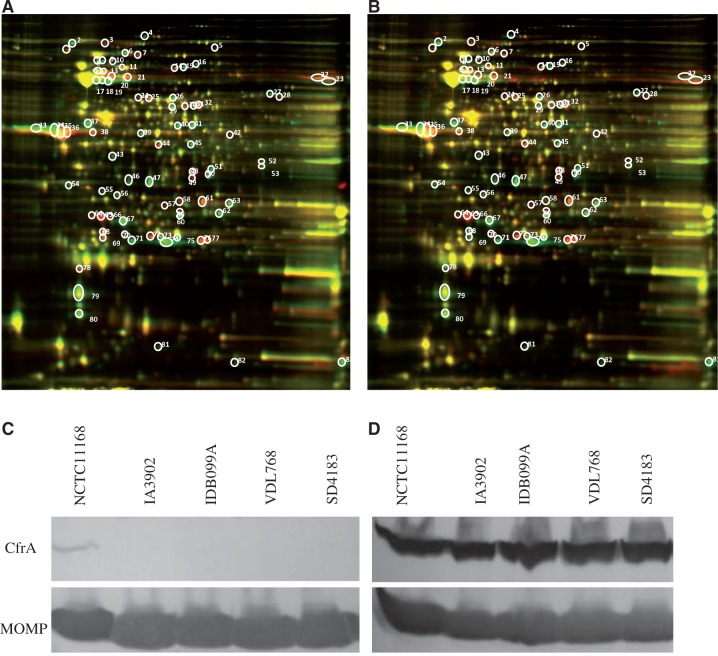
Comparative proteomics analysis of *Campylobacter jejuni* strains by 2D-DIGE and Western blotting analysis of CfrA expression. (*A*) Overlay image for in-gel comparison between IA3902 (red) and NCTC11168 (green). (*B*) Overlay image for in-gel comparison between IA5908 (red) and NCTC11168 (green). The circled and numbered protein spots in gel A and gel B indicate the differentially expressed protein spots between the strains, and those in gel A were extracted and subjected to MALDI TOF/TOF mass spectroscopy. (*C*) Western blotting analysis of the whole cell lysates of *C. jejuni* isolates grown in MH broth. (*D*) Western blotting analysis of the whole cell lysates of *C. jejuni* isolates grown in MH broth supplemented with desferrioxamine (20 µM). The CfrA protein was detected with an anti-CfrA antibody, while MOMP, which is used as an internal control, was detected with a MOMP-specific antibody.

In order to determine the differences in protein expression between IA3902 and NCTC11168, whole-cell lysates of both strains were separated by 2D-DIGE and differentially expressed protein spots were identified by MALDI-TOF MS/LC MS-MS. To enhance the identification confidence of differentially expressed proteins, another sheep abortion strain belonging to clone SA, IA5908, was included in the 2D-DIGE/mass spectrometry analysis. IA5908 was isolated from an aborted sheep placenta, and it owned the same sequence type (ST-8) as IA3902 but had a slightly different PFGE profile from IA3902 (Sahin et al. 2008). A threshold of 1.5-fold difference was applied for software DeCyder (GE Healthcare) to pick up the differentially expressed proteins. In total, 83 protein spots were found to be differentially expressed in both IA3902 and IA5908, when compared with NCTC11168 by 2D-DIGE (fig. 4*A* and *B*). Between the abortion strains IA3902 and IA5908, there were only six protein spots that showed an opposite differential expression trend (up or down) when compared with those of NCTC11168. The remaining 77 spots with the same changing trend in both IA3902 and IA5908 compared with NCTC11168 were subjected to MS identification. A total of 63 spots were identified with high confidence and represented 32 proteins (supplementary table S6, Supplementary Material online), of which 16 were ascertained to be truly differentially expressed between IA3902 and NCTC11168, with 5 over-expressed and 11 down-expressed in IA3902 (supplementary table S7, Supplementary Material online). The remaining 16 different protein spots were found to be due to sequence polymorphisms that resulted in shifts in size or isoelectric point (pI) of the homologous proteins between IA3902 and NCTC11168. It is also possible that some of these spots represent modified isoforms of the same protein, with varied abundance of different isoforms in the two strains. Flagellin is an example of this type of protein. The FlaA sequence is highly variable among *C. jejuni* isolates (Wassenaar et al. 1995) and the attached glycan moieties are also heterogeneous among isolates (McNally et al. 2006), which result in multiple isoform spots of FlaA on 2D electrophoresis. Besides the well-known examples of MOMP and FlaA, IlvE, AphC, Cj0355c (CosR, an oxidative stress response regulator), OorC, Cj1419c, Cj1110c, TrxB, GroEL, and Pta were also found to have multiple isoforms in this study (supplementary table S6, Supplementary Material online). CJSA_0596 (prophage Lp2 protein 6), FolD, PseC, and CJSA_1350 (putative methyltransferase) were shown to be truly overexpressed in IA3902, whereas ZnuA, TrxB, Tuf, CfrA, CJSA_1052 (MCP-type signal transduction protein), Tsf, GroEL, CJSA_1351 (putative methyltransferase), Cj1429c (hypothetical protein), ChuA, ChuZ (CJSA_1525), and P19 were shown to be truly down-expressed (supplementary table S7, Supplementary Material online) in IA3902. Overall, most of the differentially expressed proteins as revealed by the proteomic analysis also showed the same trend (up/down regulation) as determined by the microarray gene expression data (supplementary table S7, Supplementary Material online).

Among the differentially expressed proteins, four were due to absence or presence of their coding genes in one strain compared with the other one (supplementary table S3, Supplementary Material online). These included IA3902-specific prophage Lp2 protein 6 (CJSA_0596) and a hypothetical protein (pVir0009), and NCTC11168-specific putative MCP-type signal transduction protein (Cj1110c) and a hypothetical protein (Cj1429c).

The up-regulated protein FolD in IA3902 (CJSA_0810) was shown to be involved in the folate metabolism of *Escherichia coli* (Dari and Rabinowitz 1991). Protein PseC (CJSA_1232; upregulated in IA3902) is involved in pseudoaminic acid biosynthesis for flagellin glycosylation in *C. jejuni* (Schoenhofen et al. 2006). Gene *cjsa_1350* (upregulated in IA3902) is located within the capsule biosynthesis locus (Gundogdu et al. 2007) and contains a domain conserved among several ubiquinone/menaquinone (coenzyme metabolism) methyltransferase proteins, but its precise function is unknown.

Among the down-regulated proteins in IA3902 are translation factors TuF and TsF, and two proteins (TrxB and GroEL) belonging to the COG category of “posttranslational modification, protein turnover and chaperones.” ZnuA is the periplasmic component of a putative zinc ABC transport system and essential for *C. jejuni* growth in zinc-limiting media (Davis et al. 2009). *cjsa*_1351 (*cj1420c*) carrying a homopolymeric guanine tract encodes a putative methyltransferase, which is predicted to be functionally close to the up-regulated gene *cjsa_1350* (*cj1419c*) located within the capsule locus. Consistent with the microarray data, four proteins including CfrA, ChuA, ChuZ, and P19 associated with iron uptake and utilization were shown to be down-regulated in IA3902. CfrA (CJSA_0711) is the ferri-enterobactin receptor (Zeng et al. 2009); ChuA (CJSA_1526) is the heme-containing compound receptor and ChuZ (CJSA_1525) encodes an iron-responsive cellular hemoxygenase (Ridley et al. 2006); and P19 (CJSA_1570) is a periplasmic binding protein which mediates iron acquisition from the fungal hydroxamate siderophore ferri-rhodotorulic acid (Janvier et al. 1998). Gene mutagenesis studies showed that *cfrA*, *chuA*, and *p19* are essential for scavenging iron from exogenous siderophores enterochelin, heme, and ferri-rhodotorulic acid, respectively, in *C. jejuni* (Miller et al. 2009).

To further confirm the down-regulation of the iron-uptake systems in the isolates of clone SA, we performed immunoblotting analysis of CfrA in several *C. jejuni* isolates grown under two conditions: conventional MH media and iron-limited MH media. In conventional MH broth, CfrA was detected as a distinct band in NCTC11168 but was barely observed in the clinical abortion isolates including IA3902, 1DB099A, VDL768, and SD4183 (fig. 4). This finding is consistent with the results of transcriptomics and proteomics. When these isolates were grown in iron-limited media, CfrA was strongly induced, but there was no difference in the level produced between NCTC11168 and the abortifacient isolates, suggesting that despite the difference in basal-level expression in MH broth, induction of CfrA by iron limitation functioned similarly in all of the isolates.

## Discussion

In this study, we conducted comparative and functional genomics analyses of a highly abortifacient *C. jejuni* strain (IA3902) in comparison with *C. jejuni* NCTC11168. Both strains are able to colonize the intestinal tract, but only IA3902 is abortifacient in pregnant animals (Burrough et al. 2009). Despite their distinct pathogenic phenotypes, the genomes of the two strains are highly syntenic. Genomic sequence analysis revealed that IA3902 does not harbor pathogenicity islands or known virulence factors that may explain its highly abortifacient pathotype. However, comparative genomic analysis identified a large number of SNPs as well as indels throughout the chromosome between IA3902 and NCTC11168 (both within coding and intergenic regions), suggesting that modest genetic changes may account for the enhanced virulence of IA3902. Indeed, functional genomics approaches revealed divergent transcriptomic and proteomic profiles between IA3902 and NCTC11168, suggesting that the small genomic changes significantly influence gene/protein expression patterns and consequently the virulence phenotype in abortion induction.

Comparative genomics indicated that the most striking differences between the genomes of IA3902 and NCTC11168 were located within the loci encoding genes involved in biosynthesis and post-translational modification of capsule and flagellin (O-linked glycosylation). Capsule is a well-known virulence factor in many bacterial pathogens and was demonstrated to play a role in serum resistance and colonization in *C. jejuni*, though its exact role in the *C. jejuni* disease process has not been well defined (Guerry et al. 2012). Capsule genes in the genome of *C. jejuni* are organized into three regions, where the conserved regions 1 and 3 are involved in capsule assembly and transport, while VR2 encodes genes responsible for the synthesis of specific polysaccharides and thus determines the sugar composition and linkage (Guerry et al. 2012). Of the genes within VR11, 14 of the IA3902-specific ones are located in capsule region 2 and have no homologs in NCTC11168 (supplementary table S3, Supplementary Material online; 10 of these genes have homologs in *C. jejuni* subsp*. doylei* 269.97 and 4 of them have homologs in *C. jejuni* strain G1). The IA3902-specific genes listed within VR10 are part of the region of the genome encoding the flagellin modification (O-linked glycosylation) system. Two of them (*cjsa_1259* and *cjsa_1260*) were annotated as encoding methyltransferase domain-containing protein and one (*cjsa_1261*) as encoding hypothetical protein (supplementary table S3, Supplementary Material online), but their counterparts in NCTC11168 (*cj1321-1324*) were annotated as encoding acetyl transferases and hydroxyacyl dehydrogenases (Howard et al. 2009). Flagellin glycosylation has been shown to influence the immunogenicity, serospecificity, interaction with eukaryotic cells, and host cell specificity (Howard et al. 2009). These IA3902-specific genes in the flagellin modification locus, similar to those in capsule region, have close homologs in *C. jejuni* subsp. *doylei* strain 269.97, a subspecies of *C. jejuni* that is occasionally isolated from human clinical samples and is commonly associated with bacteremia (Lastovica 1996; Morey 1996; Lastovica and Skirrow 2000; Parker et al. 2007). Systemic infection and bacteremia are also common features of *C. jejuni* strain G1 (obtained from a patient with Guillain-Barré syndrome) (Linton et al. 2000), in which almost identical copies of four IA3902-specific capsule genes (*cjsa_1364-1367*) were present. Interestingly, the Penner serotype of IA3902 (HS:1,8) (Sahin et al. 2012) is a mosaic of those of G1 (HS:1) and *C. doylei* 269.97 (HS:17, whose capsule locus is virtually identical to that of HS:8) (Karlyshev et al. 2000; Miller et al. 2007). Although bacteremia and systemic infection seem to be the common traits of *C. jejuni* strains IA3902, G1, and *C. doylei* strain 269.97, contribution of the unique capsule and flagellin modification genes to the disease phenotype awaits to be ascertained.

Other noticeable gene-level differences in the VRs include the presence of *cjsa*_*0033*, *tetO*, *fspA2*, and *cjsa*_*1098* in IA3902. *tetO* is integrated into the chromosome and encodes resistance to tetracycline in IA3902. This insertion was confirmed by PCR to be present in all of the examined isolates of clone SA. In *Campylobacter*, *tetO* is the only *tet* gene for tetracycline resistance and is usually carried on plasmids (Gibreel et al. 2004; Poly et al. 2008). Tetracycline is the only antibiotic class currently approved in the U.S. for the treatment of *Campylobacter* abortion in sheep. Chromosomal integration of *tetO* could confer a more stable and transmissible (via natural transformation) antimicrobial resistance phenotype for clone SA, facilitating its persistence in the sheep production environment. Another noteworthy difference is the presence of a different allele of gene *fspA* in IA3902. Presence of this gene is variable among *C. jejuni* strains, and its product is secreted through the flagellar system (Poly et al. 2007). However, only the *fspA2* variant, which is present in IA3902, has been shown to be associated with apoptosis (Poly et al. 2007). *cjsa_0033* encodes a putative cytoplasmic protein with an ABC transporter nucleotide-binding domain, and *cjsa_1098* encodes a hypothetical protein carrying a twin-arginine translocation pathway signal, but their functions are currently unknown. In addition to chromosome-level differences, IA3902 carries a plasmid, named *pVir*, which is highly homologous to a previously reported plasmid from various *C. jejuni* strains. The *pVir* plasmid encodes a putative type IV secretion system and has been previously shown to be involved in the virulence of a subset of *C. jejuni* strains (Bacon et al. 2000; Bacon et al. 2002). However, PCR and DNA microarray hybridization experiments indicated that this plasmid was absent in other clone SA isolates (data not shown), suggesting that *pVir*, although present in IA3902, is not required for abortion induction.

Despite the high genomic similarity between IA3902 and NCTC11168, the global gene expression profiles between these two strains are significantly different. At the RNA level, 189 genes were detected to be differentially expressed by microarray technology; and at the protein level, 16 proteins were detected to be differentially expressed by 2D-DIGE. This finding was not surprising as small-scale changes in genomic DNA (e.g., point mutations, indels, and frameshift mutations) can have profound effects on gene expression levels (Gaynor et al. 2004; Malik-Kale et al. 2007). Although microarray and 2D-DIGE showed different levels of sensitivity, most of the differentially expressed genes identified by proteomics also showed the same trend of change by transcriptomics. In particular, the iron uptake systems were detected to be down-expressed at both the protein and RNA levels in IA3902. Although iron-associated genes may vary from strains to strains, comparative genomics indicates that both IA3902 and NCTC11168 have the same gene sets responsible for iron uptake. Both strains possess the pathways for the import of ferrous iron and iron-containing complexes such as ferric-enterochelin, ferric-rhodotorulic acid, heme, and ferri-transferrin/lactoferrin. With the exception of the ferrous iron uptake pathway, all the other pathways of ferric iron uptake were detected to be down-regulated in IA3902. The microarray and proteomic data were further supported by immunoblotting analysis of the CfrA protein of both clone SA isolates and NCTC1118 grown in regular MH broth, which revealed that the basal-level expression of CfrA in MH broth was much lower in clone SA than in NCTC11168, but the two systems are fully inducible under iron-limited conditions.

Iron affects the physiology of bacteria in two different ways: as a micronutrient for bacterial growth and as a catalyst for the formation of peroxides and hydroxyl radicals (Palyada et al. 2004). Within *C. jejuni*, one of the key roles for iron is as part of iron–sulfur complexes. These complexes are located in the active sites of several key enzymes within the metabolic networks such as the electron transport chain, amino acid metabolism, organic acid metabolism, and the citric acid cycle (Stahl et al. 2012). Without these proteins, many key metabolic pathways of *C. jejuni* would cease to function (Miller et al. 2009). On the other hand, excess iron would stimulate the formation of peroxides and hydroxyl radicals, which in turn damages the iron–sulfur complexes and DNA and kills bacterial cells (Flint et al. 1993). Thus, iron acquisition is under strict regulation to maintain a balance of intracellular concentration. Considering that the expression of the iron uptake systems is inhibited by intracellular iron concentration, their reduced basal expression (in MH broth) in IA3902 and their full induction under iron-limited conditions suggest that intracellular accumulation of iron in this strain is more efficient than in NCTC11168.

In *Campylobacter*, two regulators Fur and PerR were reported to control the gene expression of ferric iron uptake systems (Miller et al. 2009; Palyada et al. 2009). The regulatory protein Fur is primarily responsible for the regulation of genes involved in iron uptake. PerR is primarily responsible for the regulation of genes involved in oxidative stress response and resistance, but there is some overlap between the PerR and Fur regulons (Palyada et al. 2009). However, no significant expression differences of *perR* and *fur* were detected by either microarray or 2D-DIGE. *fur* expression in *C. jejuni* is controlled by two promoters located in front of the first and second ORFs upstream of *fur* (van Vliet et al. 2000). No nucleotide sequence difference is present in the two promoter regions between IA3902 and NCTC11168. Thus, the mechanisms responsible for the down-regulation of iron uptake systems in IA3902 remains unknown and to be examined in future studies.

In *C. jejuni*, the highly branched respiratory chains allow the organism to grow under microaerobic and oxygen-limited conditions (Weingarten et al. 2008). Oxygen is the main terminal electron acceptor and aerobic respiration supplies the main energy source for growth and motility, whereas anaerobic respiration with fumarate, nitrate, nitrite, and N- or S-oxides as alternative electron acceptors enables *C. jejuni* to grow under oxygen-limited conditions (Weingarten et al. 2008). *C. jejuni* is also able to utilize a variety of electron donors (Weerakoon et al. 2009). Typically, 2-oxoglutarate:acceptor oxidoreductase (Oor) has been shown to oxidize α-ketoglutarate and provide the electrons to Complex I of respiration chains (Weerakoon and Olson 2008), and hydrogenase and formate dehydrogenase oxidize hydrogen and formate as electron donors, respectively (Weerakoon et al. 2009). Compared with NCTC11168, IA3902 shows higher expression levels of electron donor enzymes and genes involved in the pathways of anaerobic respiration (supplementary table S4, Supplementary Material online). The proton pump Complex I; the reductases required for fumarate, nitrate, and nitrite as terminal electron acceptors; and the electron donor reductases including hydrogenase, formate dehydrogenase, and 2-oxoglutarate:acceptor oxidoreductase (Oor) were all up-regulated in IA3902. Additionally, the succinate-fumarate antiporter *dcuB* required for fumarate respiration was up-regulated in IA3902. As a whole, global gene expression profiling suggests that strain IA3902 is more potent in energy generation and in accommodating the lower-oxygen containing microaerobic and largely anaerobic environments, which are commonly encountered during infection of animal hosts. Compatible with the high potential of energy generation, motility genes were up-regulated in IA3902. Motility of *C. jejuni* is carried out by flagella and driven by a proton motive force from the electron transport chain and is absolutely required for colonization and disease progression. A number of flagellar export apparatus genes including the hook and basal body structure genes and the energy taxis genes were expressed significantly higher in IA3902 than in NCTC11168. Motility agar assay also demonstrated that IA3902 is more motile than NCTC11168 (data not shown). These results suggest that upregulation of metabolism and motility may be a factor for the enhanced virulence of IA3902.

Another striking difference is in the potential of carbon source utilization between IA3902 and NCTC11168. Although both IA3902 and NCTC11168 harbor similar pathways for metabolizing serine, proline, glutamate, aspartate, and lactate, the sheep abortion strain IA3902 may be more capable in utilizing of serine and less capable in utilizing proline, glutamate, aspartate, and lactate, as suggested by the microarray data (supplementary table S4, Supplementary Material online). *C. jejuni* strains have been shown to exhibit considerable metabolic diversity in their ability to utilize a range of carbon sources (Hofreuter et al. 2008; Stahl et al. 2011). Different capability of carbon source utilization could affect the potential to colonize different tissues and organs by *C. jejuni* during infection (Hofreuter et al. 2008). Previous studies have shown that serine, proline, glutamate, and aspartate are preferentially used as carbon and energy sources for *C. jejuni* growth in vitro (Leach et al. 1997; Velayudhan et al. 2004; Leon-Kempis Mdel et al. 2006) and that serine catabolism as well as transporters are absolutely critical for host colonization (Hendrixson and DiRita 2004; Velayudhan et al. 2004). The up-regulated pathway of serine utilization may enhance the overall ability of IA3902 to infect an animal host, but whether the preferential utilizations of certain amino acids would benefit the growth of *C. jejuni* in a specific host tissue (such as placenta) is unknown.

These findings suggest that a set of differentially expressed genes or a unique gene expression pattern (i.e., upregulation in the pathways of energy generation, respiration, motility, and serine utilization; downregulation in the pathways of iron uptake and utilization) may be associated with high virulence (and predominance) of clone SA in *Campylobacter*-induced sheep abortion in the United States. Although many of the genes exhibiting significant expression differences are functionally related, they are physically scattered throughout the genome, suggesting that these two strains may possess considerable differences in global regulation. Given that the gene contents are highly similar, the numerous SNPs could be the contributor of global gene expression changes.

In conclusion, the genome of IA3902 is highly similar and syntenic with the non-abortion strain NCTC11168, and 12 VRs and several thousand SNPs were identified between the two strains. Several genes involved in capsule biosynthesis and modification as well as flagellin O-linked glycosylation are distinct for IA3902 and are closely related to homologs in *C. jejuni* subsp. *doylei* and *C. jejuni* G1, which were reportedly associated more often with bacteremia and systemic infections. Transcriptomic and proteomic comparions uncovered significant difference in the gene expression profiles. Specifically, the pathways of energy generation, respiration, motility, and serine utilization were upregulated, whereas the pathways for iron uptake and utilization of proline, glutamate, aspartate, and lactate were down-regulated. Collectively, these differences in gene contents and expressions may contribute to the hyper-virulence of *C. jejuni* clone SA in inducing abortion. These findings shed light on the pathogenesis of *C. jejuni* clone SA and provide directions for understanding specific virulence mechanisms (table 1).

**Table 1:** Key Features of *C. jejuni* IA3902 Revealed by Combined Analysis of the Genomic, Transcriptomic and Proteomic Data

	Features of IA3902 Revealed by Muti-omics Approaches	Potential Implication for IA3902 Pathogenesis
Comparative genomics	Unique genes in O-linked glycosylation	Flagellar modification, host cell specificity, colonization, and translocation
	Unique genes in capsule biosynthesis and modification	Serum resistance and bacteremia induction
	*fspA2*	Tissue damage
	*tetO*	Antibiotics resistance and persistence
	SNPs and indels	Global regulation of gene expression

Transcriptomics	↓ Iron uptake systems	Adaptation to iron-limited condition
	↑ Energy production and conversion	Motility, colonization, and translocation
	↑ Cell motility	
	↑ Anaerobic respiration	Adaptation to oxygen limitation
	↑ Serine utilization	Selective utilization of amino acid and growth in different tissues
	↓ Proline, glutamate, aspartate, and lactate utilization	

Proteomics	↓ P19	Adaptation to iron-limited condition
	↓ CfrA	
	↓ ChuZ	
	↓ChuA	
	↑ PseC	Flagellar modification and host cell specificity

Note.—Genes with increased mRNA or protein expression in IA3902 are indicated by upward arrows and those with decreased mRNA or protein expression in IA3902 are indicated by downward arrows.

## Supplementary Material

Supplementary figures S1–S3 and tables S1–S7 are available at *Genome Biology and Evolution* online (http://www.gbe.oxfordjournals.org/).

Supplementary Data
