# Supplementary material for: Multi-omics Approaches to Deciphering a Hypervirulent Strain of Campylobacter jejuni
Source: Genome Biol Evol. 2013 Nov 6;5(11):2217–30. doi: 10.1093/gbe/evt172 (PMC3845652; doi:10.1093/gbe/evt172)
Supplement: Supplementary Data [file supp_5_11_2217__index.html]

Multi-omics approaches to deciphering a hypervirulent strain of Campylobacter jejuni — Multi-omics Approaches to Deciphering a Hypervirulent Strain of Campylobacter jejuni — Supplementary Data 

# Multi-omics Approaches to Deciphering a Hypervirulent Strain of *Campylobacter jejuni*

## Supplementary Data

files

**Files in this Data Supplement:**

- Supplementary Data - pdf file
