# Supplementary material for: Multi-omics Approaches to Deciphering a Hypervirulent Strain of Campylobacter jejuni
Source: Genome Biol Evol. 2013 Nov 6;5(11):2217–30. doi: 10.1093/gbe/evt172 (PMC3845652; doi:10.1093/gbe/evt172)
Supplement: Supplementary Data [file supp_evt172_Supplemental_material.pdf]

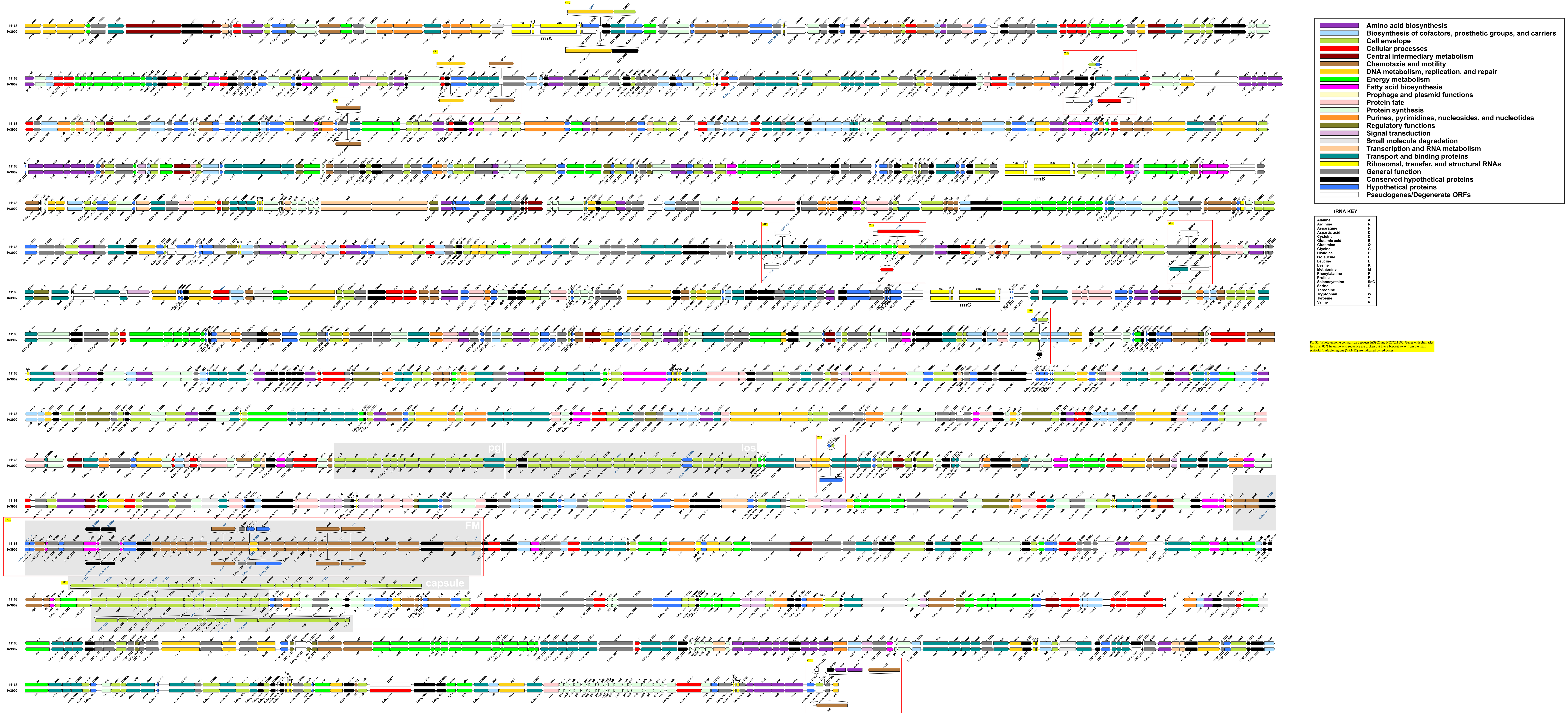

Fig S1: Whole-genome comparison between IA3902 and NCTC11168. Genes with similarity less than 85% in amino acid sequence are broken out into a bracket away from the main scaffold. Variable regions (VR1-12) are indicated by red boxes.

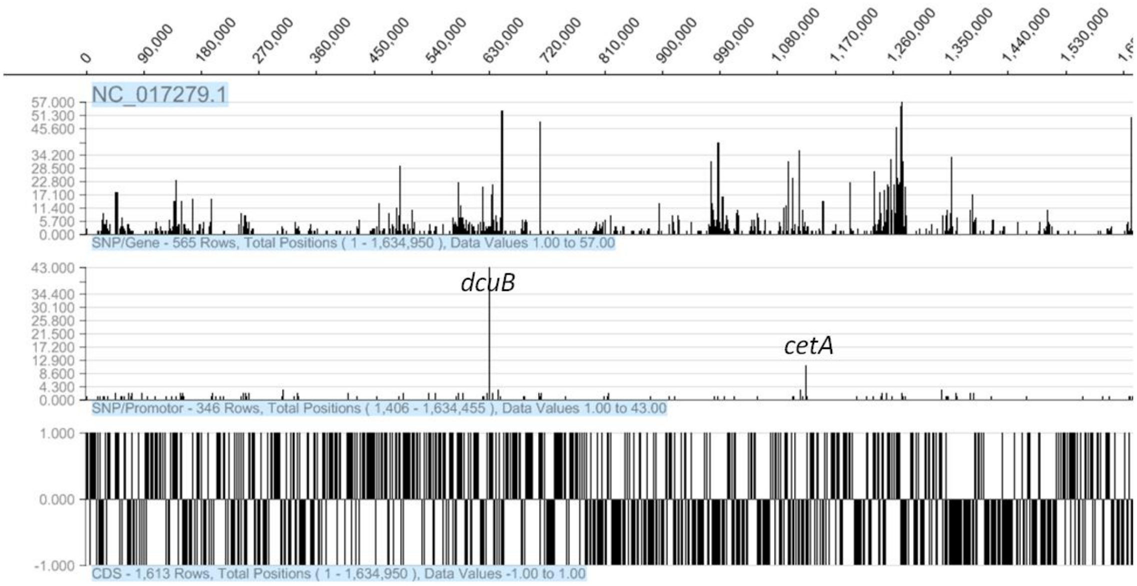

A

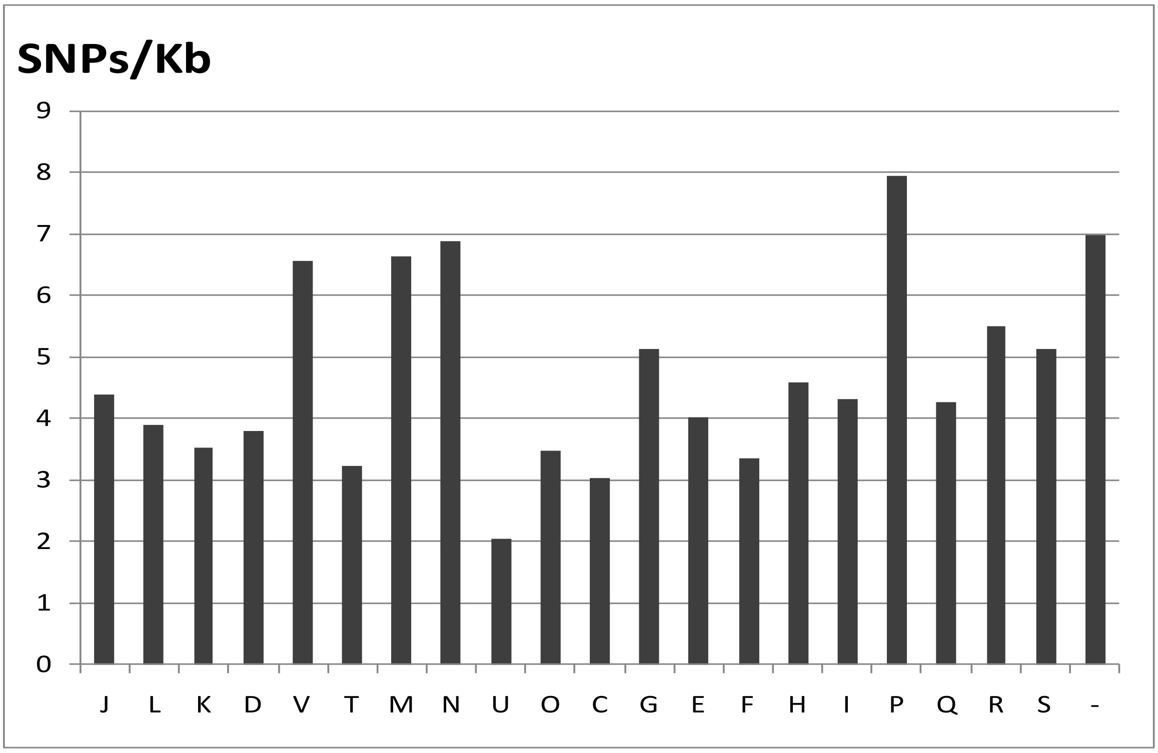

B

Fig S2. **(A)** The distribution of SNPs between the genomes of *C. jejuni* IA3902 and NCTC11168. The first track shows the nonsynonymous SNPs for each gene. The second track shows the intergenic SNPs for promoter region of each gene. The third track depicts the CDSs of IA3902. **(B)** The average numbers of nonsynonymous SNPs per Kb for each gene in different COG categories. COG category codes: J, Translation;

L, Replication, recombination and repair; K, Transcription; D, Cell cycle control, mitosis and meiosis; V, Defense mechanisms; T, Signal transduction mechanisms; M, Cell wall/membrane biogenesis; N, Cell motility; U, Intracellular trafficking and secretion; O, Posttranslational modification, protein turnover, chaperones; C, Energy production and conversion; G, Carbohydrate transport and metabolism; E, Amino acid transport and metabolism; F, Nucleotide transport and metabolism; H, Coenzyme transport and metabolism; I, Lipid transport and metabolism; P, Inorganic ion transport and metabolism; Q, Secondary metabolites biosynthesis, transport and catabolism; R, General function prediction only; S, Function unknown; and -, Not in COGs.

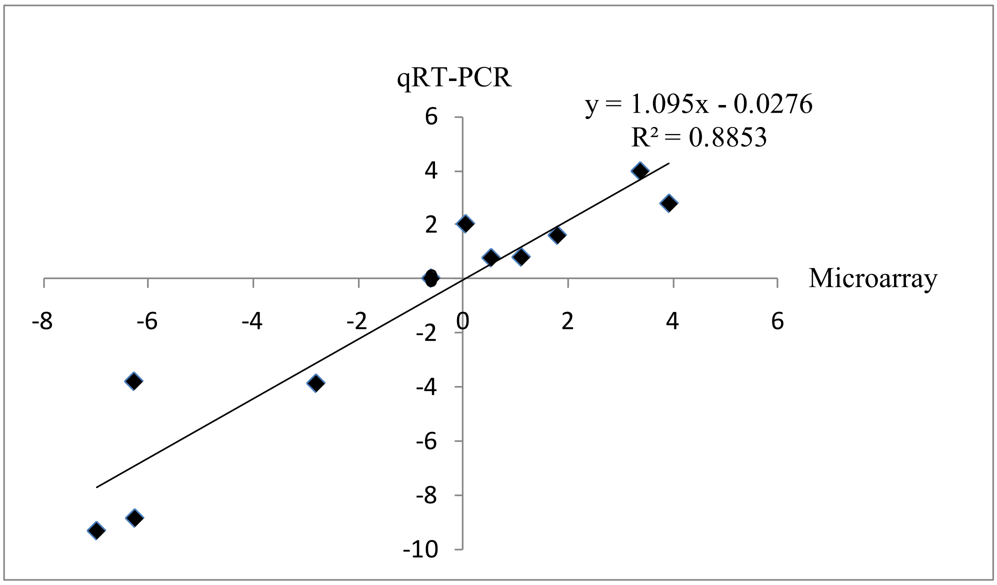

Fig. S3. Validation of microarray data using quantitative real-time RT-PCR. The transcriptional levels for the selected 11 genes were determined by quantitative real-time RT-PCR. Four up-regulated genes, four down-regulated genes and three genes that did not show significant variation in the microarray experiments are presented. Mean log2 ratios obtained in qRT-PCR experiments are plotted against the mean log2 ratios obtained with the microarrays. The high correlation coefficient values (R^2) indicated that the microarray data was highly reliable in this study.

Table S1. Sequencing and assembly statistics of *C. jejuni* IA3902 provided by 454 Life Sciences

Total number of reads	361,002
Total number of bases	101,279,741
Average read length	280.6
No. of total bases in all contigs	1,651,980
No. of total bases in large contigs	1,650,004
No. of all contigs	37
No. of large contigs	30
Size (bp) of large contigs	518 to 481,571
Size (bp) of small contigs	122 to 365
N50 contig size[a]	182,457
Q40+bases[b]	1,649,311
Average coverage depth fold	55.7

[a]The N50 size is the length such that 50% of the assembled genome lies in contigs of N50 size or longer
[b]Q40+ bases: This is all the bases in the assembled contigs that carry a phred-equivalent quality score of 40 and above. A score of 40 is equivalent to an error rate of 10^{-4}, or an accuracy of 99.99%.

Table S2. General and Genome properties of *C. jejuni* IA3902

General		
Serotype	HS:1,8	
MLST	ST-8/CC-21	
PFGE	DBRS16.008/DBRK02.0028	
FlaA SVR	Allele type 122/peptide type 4	
Susceptibility pattern	Tet^R, Pen^R, Enr^S, Tyl^S, Flor^S, Kan^S,	
**Genome**	**chromosome**	**plasmid**
Size (bp)	1,635,045	37,174
% CG content	30.57	25.91
CDS numbers	1613	53
Assigned function	1378 (85.5%)	15
hypothetical/conserved	233 (14.5%)	38
% Coding	92.9	86.8
Avg. length (bp)	942	576
Pseudogenes	20	0
tRNA	44	-
rRNA operons	3	-
miscellaneous RNA	4	-
Transposase fragments	4	-
CRISPR	4	-
PolyG:C tracts	27	1

CDS no. excludes pseudogenes.
Assigned function includes those assigned to a specific function and those assigned to a general function, and putative functions.
Average length and %Coding values are based on CDS only, exclude pseudogenes and RNA genes.
PolyG:C tracts: those of e 7 consecutive G or C residue

Table S3. List of different genes between IA3902 and NCTC11168

VRs[a]	Locus tag	Size (aa)	IA3902	NCTC 11168	Annotation	Remarks (comparisons to NCTC11168/81-176/81116/RM1221 mainly)
VR1	CJSA_0032	1257	+	+	Type II rest.mod.enzyme	Cj0031, 1243aa; 82% identity between the homologs
	CJSA_0033	708	+	-	putative cytoplasmic protein	Replaced by Cj0033, 593aa (integral membrane protein); not present in any *Campylobacter*; 49% identity to HPKB_1419, 716aa, putative cytoplasmic protein; has ABC transporter nucleotide binding domain (not a significant match); 34% identity to MimD; 22% GCcontent
VR2	CJSA_130	662	+	+	McrBC restriction endonuclease	Cj0139, 783aa; 65% identity between the homologs
	CJSA_135	651	+	+	Methyl accepting chemotaxis protein	Cj0144, 659aa; 72% identity between the homologs
VR3	CJSA_0192	61	+	-	hypothetical protein	CJJ81176_pTet0049 homolog
	CJSA_0193	639	+	-	tetracycline resistance protein TetO	CJJ81176_pTet0048 homolog
	TnpV	124	+	-	transposase V; pseudo	MGAS2096_Spy1150 homolog (*Streptococcus pyogenes*)
	Cj0201c	201	-	+	putative integral membrane protein	Totally missing in IA3902; present in other strains
	Cj0202c	113	-	+	hypothetical protein	Totally missing in IA3902; present in other strains
VR4	CJSA_0239	706	+	+	Methyl accepting chemotaxis protein	Cj0262c, 665aa; 68% identity between the homologs
VR5	CJSA_585	223	+	+	hypothetical protein	Cj0617, 203aa; 57% identity between the homologs
	CJSA_586	187	+	+	hypothetical protein	Cj0618, 215aa; 57% identity between the homologs
VR6	CJSA_0596	349	+	-	prophage Lp2 protein 6 (uncharacterized conserved protein)	Replaced by Cj0628 (CapA; autotransporter, 144aa), putative lipoprotein (high similarity to CJSA_1588 and Cj1677-CapB; both 1121aa, which does not seem transcribed or translated in many *C. jejuni*); has homolog in 81116 and 81-176. Replaced by R/M in RM1221. Neither *capA* nor *capB* is present in the other 3 strains.
VR7	CJSA_0621	517	+	-	MFS di-/tripeptide transporter	Has homologs in other *C. jejuni* strains; missing in 11168
	CJSA_0622/3	476	+	+	MFS di-/tripeptide transporter, pseudogene	Replaced by Cj0654c, pseudogene in 11168
VR8	CJSA_0814	142	+	-	FspA2 (involved in in-vitro cell binding and apoptosis)	Replaced by Cj0859c, 142aa, FspA1 (both are *Campylobacter jejuni*-specific virulence factor that is secreted through the flagella filament). Identical to that of strain 84-25 and few other strains (ATCC43430-from calf feces-).
	Cj0860	290	-	+	putative integral membrane protein	Totally missing in IA3902; present in some other *C. jejuni* strains (mainly fspA1), but missing in many C. jejuni strains.
	CJSA_1098	633	+	-	TAT pathway signal protein	Has identical in CJ84-25 (same neighbors too); homologs only in few other

						jejuni and *C. lari*, and many other bacteria. Has significant Pfam match to NAD(P)-binding Rossmann-like domain.
VR9	Cj1158c	75	-	+	small hydrophobic protein	These 3 ORFs replaced by CJSA_1098 in IA3902
	Cj1159c	40	-	+	small hydrophobic protein	
	Cj1160c	59	-	+	hypothetical protein	
VR10	CJSA_1243	404	+	+	hypothetical protein	Cj1305c, 405aa; 82% identity between the homologs
	CJSA_1244	402	+	+	hypothetical protein	Cj1306c, 408aa; 76% identity between the homologs
	CJSA_1256	650	+	+	Motility accessory factor 1	Cj1318, 645aa; 81% identity between the homologs
	CJSA_1259	243	+	-	methyltransferase domain-containing protein	Replaced by Cj1321; part of O-linked glycosylation locus; has close homology in C. doylei only
	CJSA_1260	235	+	-	methyltransferase domain-containing protein	Replaced by Cj1322; part of O-linked glycosylation locus; has homologs in C. upsaliensis and doylei.
	CJSA_1261	705	+	-	hypothetical protein	Replaced by Cj1323/24; part of O-linked glycosylation locus; represented by multiple ORFs in other strains
	CJSA_1269	650	+	+	PseD	Cj1333; 84% identity between the homologs; O-linked locus
	CJSA_1271	650	+	+	Maf4	Cj1335; 81% identity between the homologs; O-linked locus
VR11	CJSA_1352	612	+	+	Putative sugar transferase	Cj1421c (612aa), Cj1422c (625aa); 56% and 50% identity between the homologs; CPS locus
	CJSA_1353	240	+	-	CPS biosynthesis protein	Has homolog only in *C. doylei* 269.97 (JJD26997_1800); isolated from human blood; HS:17; CPS locus
	CJSA_1354	507	+	-	CPS biosynthesis protein	Has homolog only in *C. doylei*; CPS locus
	CJSA_1355	132	+	-	capsule biosynthesis phosphatase	Has homolog only in *C. doylei*; CPS locus
	CJSA_1356	639	+	-	putative CPS biosynthesis protein	No homologs; CPS locus
	CJSA_1357	619	+	-	putative sugar nucleotidyltransferase	No homologs; CPS locus
	CJSA_1358	101	+	-	hypothetical protein	Has homolog only in *C. doylei*; CPS locus
	CJSA_1359	111	+	-	hypothetical protein	Has homolog only in *C. doylei*; CPS locus
	CJSA_1360	241	+	-	putative nucleotidyl transferase	Has homolog only in *C. doylei*; CPS locus
	CJSA_1361	212	+	-	HAD superfamily hydrolase	Has homolog only in *C. doylei*; CPS locus
	CJSA_1362	212	+	-	hypothetical protein	Has homolog only in *C. doylei*; CPS locus
	CJSA_1363	637	+	+	putative sugar transferase	Cj1421c (612aa); Cj1422 (625aa) 48% identity between homologs; CPS locus
	CJSA_1364	851	+	-	putative sugar transferase	Very similar to *C. jejuni* G1 (HS1.08; GBS isolate); CPS locus
	CJSA_1365	1095	+	-	TagF	Identical to *C. jejuni* G1; CPS locus
	CJSA_1366	402	+	-	Conserved hypothetical protein	Identical to *C. jejuni* G1; CPS locus

	CJSA_1367	315	+	-	TagD	Identical to *C. jejuni* G1; CPS locus
	Cj1423c	221	-	+	hddC	Present in some C. jejuni; CPS locus
	Cj1424c	201	-	+	gmhA2	Present in some C. jejuni
	Cj1425c	339	-	+	hddA	Present in some C. jejuni
	Cj1426c	283	-	+	putative methyltransferase family protein	Present in some C. jejuni
	Cj1427c	313	-	+	putative sugar-nucleotide epimerase/dehydratease	Present in some *C. jejuni*
	Cj1428c	346	-	+	GDP-L-fucose synthetase	Present in some *C. jejuni*
	Cj1429c	308	-	+	hypothetical protein	Present in some C. jejuni
	Cj1430c	181	-	+	rfbC	Present in some *C. jejuni*
	Cj1431c	582	-	+	hddD	Present in some *C. jejuni*
	Cj1432c	1031	-	+	putative sugar transferase	Present in some *C. jejuni*
	Cj1433c	368	-	+	hypothetical protein	Present in some *C. jejuni*
	Cj1434c	445	-	+	putative sugar transferase	Present in some *C. jejuni*
	Cj1435c	211	-	+	putative phosphatase	Present in some *C. jejuni*
	Cj1436c	390	-	+	aminotransferase	Present in some *C. jejuni*
	Cj1437c	367	-	+	aminotransferase	Present in some *C. jejuni*
	Cj1438c	776	-	+	putative sugar transferase	Present in some *C. jejuni*
	Cj1439c	368	-	+	glf	Present in some *C. jejuni*
	Cj1440c	407	-	+	putative sugar transferase	Present in some *C. jejuni*
	Cj1441c	393	-	+	kfiD	Present in some *C. jejuni*
	Cj1442c	544	-	+	putative sugar transferase	Present in some *C. jejuni*; CPS locus
VR12	Cj1722c	33	-	+	hypothetical protein	Not present in any other *C. jejuni* strains
	Cj1723c	74	-	+	putative periplasmic protein	
	Cj1725	199	-	+	putative periplasmic protein	Not present in *C. jejuni* 81116, either (seems like they are present in most other strains).
	Cj1726c	293	-	+	homoserine O-succinyltransferase	
	Cj1727c	423	-	+	putative O-acetylhomoserine-lyase	
	Cj1728c	51	-	+	small hydrophobic protein	Present in 81-176, too.
	CJSA_1632	852	+	+	FlgE2	Cj1729c, 865aa, 80% identity between the homologs

**Pseudogenes**[b]						
**Functional in IA3902**	CJSA_0491	589	+	-	Na/Pi-cotransporter, putative	Cj0522/3/4 pseudogene in NCTC11168
	CJSA_0639	96	+	-	putative potassium-transporting ATPase subunit B N-terminus	Cj0678 pseudogene in NCTC11168
	CJSA_0771	174	+	-	hypothetical protein	Cj0815/6 pseudogene in NCTC11168
	CJSA_1007	206	+	-	nitroreductase family protein	Pseudogene in 11168 (Cj1064) and RM1221; present in 81-176 and CJ84-25; divided in two ORFs in 81116. A similar intact gene present immediately downstream in all strains!
	CJSA_1393	393	+	-	general secretory pathway protein F	Pseudogene in 11168 (Cj1470c); intact in other *C. jejuni* strains
	CJSA_1474	107	+	-	arsenical resistance operon repressor (ArsR)	Replaced by Cj1561/62, pseudogene in NCTC11168, 59/50aa, putative transcriptional regulator; homologs present in RM1221/81116
	CJSA1588	1121	+	-	putative lipoprotein	Cj1677, pseudogene in NCTC11168
**Functional in NCTC11168**	Cj0030	465	-	+	hypothetical protein	CJSA_0030/31 pseudogene in IA3902
	Cj0170	247	-	+	hypothetical protein	CJSA_0160/161, pseudogene in IA3902
	Cj0203	448	-	+	putative citrate transporter	Interrupted by insertion of a 5425bp region in IA3902; pseudo in IA3902 (CJSA_0191).
	Cj0484	410	-	+	putative MFS (Major Facilitator Superfamily) transport protein	CJSA_0454/5, pseudogene in IA3902
	Cj0566	488	-	+	hypothetical protein	CJSA_0533, pseudogene in IA3902
	Cj0570	355	-	+	putative ATP/GTP binding protein	CJSA_0537/8, pseudogene in IA3902
	Cj0672	62	-	+	putative periplasmic protein	Replaced by CJSA_0636, pseudogene in IA390262aa
	Cj0677	681	-	+	potassium-transporting ATPase subunit B	CJSA_0638, pseudogene in IA3902
	Cj0679	606	-	+	truncated KdpD protein	CJSA_0640, pseudogene in IA3902
	Cj1110c	429	-	+	MCP-type signal transduction protein	Pseudogene in IA3902 only (CJSA_1052); One study show it did not affect chick colonization; one study found it is membrane associated; much lower expression level in 3902
	Cj1631c	289	-	+	conserved hypothetical protein	Pseudogene in IA3902 (CJSA_1543); intact in other *C. jejuni* strains
	Cj1721c	214	-	+	putative outer membrane protein	Pseudogene in IA3902 (CJSA_1630); intact in other *C. jejuni*

**pVir (compared to pVir of strain 81-176)[c]**						
	CJSA_pVir0017	148	+	NA	conserved hypothetical protein	Replaced by CJ81176_pVir0017, hypothetical protein
	CJSA_pVir0018	145	+	NA	hypothetical protein	Replaced by CJ81176_pVir0018, hypothetical protein
	CJ81176_pVir0043	42	-	NA	hypothetical protein	None
	CJSA_pVir0045	94	+	NA	hypothetical protein	Replaced by CJ81176_pVir0047, hypothetical protein
	CJSA_pVir0046	91	+	NA	addiction module toxin	Replaced by CJ81176_pVir0047, hypothetical protein

a. The plus (+) and minus (-) indicate the presence and absence of genes, respectively.
b. Plus (+) indicates genes predicted to be functional and Minus (-) indicates genes predicted to be pseudogenes in IA3902 or NCTC11168
c. The plus (+) and minus (-)indicate the presence and absence of genes in IA3902, respectively; NA indicate the gene is not available for NCTC11168

Table S4A. Up-regulated Genes in IA3902 by microarray

Name	Gene	Annotation	Fold Change(UP)
Cj0012c	*rrc*	non-haem iron protein	10.34
Cj0043	*flgE*	flagellar hook protein	3.66
Cj0055c		conserved hypothetical protein Cj0055c	3.54
Cj0056c		hypothetical protein	7.46
Cj0122		hypothetical protein	17.86
Cj0155c	*rpmE*	50S ribosomal protein L31	2.64
Cj0224	*argC*	N-acetyl-gamma-glutamyl-phosphate reductase	4.03
Cj0225		putative acetyltransferase	4.81
Cj0226	*argB*	acetylglutamate kinase	5.33
Cj0227	*argD*	putative acetylornithine/succinyldiaminopimelate aminotransferase	4.75
Cj0239c	*iscU*	NifU protein homolog	5.23
Cj0240c	*iscS*	cysteine desulfurase (NifS protein homolog)	2.41
Cj0241c		putative iron-binding protein	2.76
Cj0301c	*modB*	putative molybdenum transport system permease protein	4.04
Cj0302c		putative molybdenum-pterin binding protein	5.51
Cj0303c	*modA*	putative molybdate-binding lipoprotein	5.67
Cj0324	*ubiE*	ubiquinone/menaquinone biosynthesis methyltransferase	3.25
Cj0333c	*fdxA*	ferredoxin	10.54
Cj0343c		putative integral membrane protein	2.35
Cj0354c	*fdxB*	ferredoxin	2.67
Cj0391c		hypothetical protein	8.27
Cj0410	*frdB*	fumarate reductase iron-sulfur subunit	3.45
Cj0414		putative oxidoreductase subunit	3.56
Cj0415		putative GMC oxidoreductase subunit	4.12
Cj0416		hypothetical protein	11.27
Cj0417		hypothetical protein	11.69
Cj0428		hypothetical protein	2.71
Cj0524		hypothetical protein	10.77
Cj0536	*oorA*	2-oxoglutarate-acceptor oxidoreductase subunit OorA	2.14
Cj0545	*hemC*	porphobilinogen deaminase	2.59
Cj0547	*flaG*	flagellar protein FlaG	6.33
Cj0548	*fliD*	flagellar capping protein	4.78
Cj0549	*fliS*	flagellar protein FliS	4.64
Cj0550		hypothetical protein	3.68
Cj0589	*ribF*	bifunctional riboflavin kinase/FMN adenylyltransferase	2.59
Cj0590		putative SAM-dependent methyltransferase	6.11
Cj0613	*pstS*	putative periplasmic phosphate binding protein	2.81
Cj0614	*pstC*	putative phosphate transport system permease protein	7.50
Cj0622	*hypF*	carbamoyltransferase	5.08
Cj0671	*dcuB*	anaerobic C4-dicarboxylate transporter	15.08
Cj0676	*kdpA*	pseudo	3.38

Cj0687c	*flgH*	flagellar basal body L-ring protein	5.02
Cj0697	*flgG2*	flagellar basal-body rod protein	3.25
Cj0698	*flgG*	flagellar basal body rod protein FlgG	2.73
Cj0769c	*flgA*	flagellar basal body P-ring biosynthesis protein FlgA	3.28
Cj0770c		putative NLPA family lipoprotein	10.56
Cj0823		hypothetical protein	2.36
Cj0851c		putative integral membrane protein	6.88
Cj0864		putative periplasmic protein	4.37
Cj0866		pseudo	3.15
Cj0873c		hypothetical protein	3.73
Cj0874c		putative cytochrome C	2.92
Cj0949c		putative peptidyl-arginine deiminase family protein	2.32
Cj0965c		putative acyl-CoA thioester hydrolase	2.31
Cj0977		hypothetical protein	8.85
Cj1001	*rpoD*	RNA polymerase sigma factor RpoD	2.06
Cj1012c		hypothetical protein	11.52
Cj1025c		hypothetical protein	6.56
Cj1026c		putative lipoprotein	2.87
Cj1042c		putative transcriptional regulatory protein	3.88
Cj1108	*clpA*	ATP-dependent Clp protease ATP-binding subunit	2.05
Cj1123c	*pglD*	acetyltransferase	2.93
Cj1169c		putative periplasmic protein	6.02
Cj1190c	*cetA*	bipartate energy taxis response protein cetA	2.43
Cj1224		putative iron-binding protein	3.92
Cj1225		conserved hypothetical protein Cj1225	4.44
Cj1253	*pnp*	polynucleotide phosphorylase/polyadenylase	2.73
Cj1262	*racS*	two-component sensor (histidine kinase)	2.13
Cj1265c	*hydC*	Ni/Fe-hydrogenase B-type cytochrome subunit	3.49
Cj1266c	*hydB*	Ni/Fe-hydrogenase large subunit	4.03
Cj1267c	*hydA*	Ni/Fe-hydrogenase small chain	2.96
Cj1288c	*gltX*	glutamyl-tRNA synthetase	2.34
Cj1290c	*accC*	biotin carboxylase	2.61
Cj1291c	*accB*	putative biotin carboxyl carrier protein of acetyl-CoA carboxylase	2.28
Cj1293	*pseB*	UDP-GlcNAc-specific C4,6 dehydratase/C5 epimerase	2.19
Cj1316c	*pseA*	pseudaminic acid biosynthesis PseA protein	5.20
Cj1334	*maf3*	motility accessory factor (function unknown)	4.70
Cj1358c	*nrfH*	putative periplasmic cytochrome C	3.17
Cj1362	*ruvB*	Holliday junction DNA helicase RuvB	3.92
Cj1464	*flgM*	hypothetical protein	8.63
Cj1465		hypothetical protein	4.63
Cj1466	*flgK*	flagellar hook-associated protein FlgK	3.29
Cj1500		putative inner membrane protein	2.66
Cj1501		hypothetical protein	2.71
Cj1507c		putative regulatory protein	2.29
Cj1509c	*fdhC*	putative formate dehydrogenase, cytochrom B subunit	2.50
Cj1510c	*fdhB*	putative formate dehydrogenase iron-sulfur subunit	3.05

Cj1511c	*fdhA*	putative formate dehydrogenase large subunit (Selenocysteine containing)	3.83
Cj1513c		possible periplasmic protein	2.46
Cj1514c		hypothetical protein	2.30
Cj1522c		putative CRISPR-associated protein	2.51
Cj1530	*coaE*	dephospho-CoA kinase	6.69
Cj1531	*dapF*	diaminopimelate epimerase	2.47
Cj1540		putative periplasmic protein	2.80
Cj1566c	*nuoN*	NADH dehydrogenase I chain N	3.03
Cj1570c	*nuoJ*	NADH dehydrogenase subunit J	2.29
Cj1572c	*nuoH*	NADH dehydrogenase subunit H	2.64
Cj1577c	*nuoC*	NADH dehydrogenase subunit C	2.30
Cj1578c	*nuoB*	NADH dehydrogenase subunit B	2.25
Cj1586	*cgb*	single domain haemoglobin	2.81
Cj1622	*ribD*	riboflavin-specific deaminase/reductase	2.44
Cj1624c	*sdaA*	L-serine dehydratase	9.27
Cj1625c	*sdaC*	amino acid transporter	10.31
Cj1632c		putative periplasmic protein	9.32
Cj1656c		hypothetical protein	7.83
Cj1685c	*bioB*	biotin synthase	3.51
Cj1686c	*topA*	DNA topoisomerase I	5.51
Cj1731c	*ruvC*	Holliday junction resolvase	2.48

Table S4B. Down-regulated genes in IA3902 by microarray

Name	Gene	Annotation	Fold Change(Down)
Cj0021c		putative fumarylacetoacetate (FAA) hydrolase family protein	12.24
Cj0074c		putative iron-sulfur protein	2.08
Cj0075c		putative oxidoreductase iron-sulfur subunit	2.02
Cj0076c	*lctP*	L-lactate permease	3.11
Cj0079c	*cdtA*	cytolethal distending toxin A	2.59
Cj0086c	*ung*	uracil-DNA glycosylase	5.76
Cj0105	*atpA*	F0F1 ATP synthase subunit alpha	2.05
Cj0109	*exbB3*	putative MotA/TolQ/ExbB proton channel family protein	2.61
Cj0141c		putative ABC transporter integral membrane protein	2.49
Cj0143c		putative periplasmic solute binding protein for ABC transport system	3.46
Cj0146c	*trxB*	thioredoxin reductase	4.68
Cj0168c		putative periplasmic protein	2.68
Cj0174c	*cfbpB*	putative iron-uptake ABC transport system permease protein	8.61
Cj0175c	*cfbpA*	putative iron-uptake ABC transport system,periplasmic iron-binding protein	7.03
Cj0176c		putative lipoprotein	7.25
Cj0182		putative transporter	2.74
Cj0203		putative citrate transporter	334.83
Cj0372		putative glutathionylspermidine synthase	2.15
Cj0375		putative lipoprotein	2.68

Cj0376		putative periplasmic protein	3.98
Cj0393c	*mqo*	putative malate:quinone oxidoreductase	3.39
Cj0481	*dapA*	putative dihydrodipicolinate synthase	16.90
Cj0482	*uxaA'*	putative altronate hydrolase N-terminus	7.34
Cj0483	*uxaA'*	putative altronate hydrolase C-terminus	21.15
Cj0484		putative MFS (Major Facilitator Superfamily) transport protein	8.25
Cj0485		short chain dehydrogenase	163.96
Cj0486		putative sugar transporter	13.13
Cj0488		conserved hypothetical protein Cj0488	7.47
Cj0489	*ald'*	putative aldehyde dehydrogenase N-terminus	6.88
Cj0552		hypothetical protein	2.10
Cj0591c		putative lipoprotein	3.15
Cj0592c		putative periplasmic protein	2.76
Cj0628		putative lipoprotein	4.29
Cj0755	*cfrA*	ferric enterobactin uptake receptor	77.88
Cj0762c	*aspB*	aspartate aminotransferase	20.91
Cj0774c		ABC transport system ATP-binding protein	2.15
Cj0778	*peb2*	major antigenic peptide PEB2	2.20
Cj0790	*purU*	formyltetrahydrofolate deformylase	2.34
Cj0818		putative lipoprotein	6.25
Cj0833c		putative oxidoreductase	3.49
Cj0834c		ankyrin repeat-containing putative periplasmic protein	4.77
Cj0835c	*acnB*	bifunctional aconitate hydratase 2/2-methylisocitrate dehydratase	5.44
Cj0900c		small hydrophobic protein	2.11
Cj0903c		putative amino-acid transport protein	2.82
Cj0909		putative periplasmic protein	2.79
Cj0910		putative periplasmic protein	2.29
Cj0916c		conserved hypothetical protein Cj0916c	2.97
Cj0919c		putative ABC-type amino-acid transporter permease protein	4.88
Cj0920c		putative ABC-type amino-acid transporter permease protein	8.33
Cj0921c	*peb1A*	bifunctional adhesin/ABC transporter aspartate/glutamate-binding protein	7.12
Cj0922c	*pebC*	ABC-type amino-acid transporter ATP-binding protein	6.33
Cj1007c		putative mechanosensitive ion channel family protein	2.24
Cj1008c	*aroB*	3-dehydroquinate synthase	2.60
Cj1102	*truB*	tRNA pseudouridine synthase B	3.12
Cj1110c		putative MCP-type signal transduction protein	25.42
Cj1137c		putative glycosyltransferase	4.45
Cj1203c		putative integral membrane protein	4.47
Cj1275c		putative peptidase M23 family protein	2.99
Cj1308		putative acyl carrier protein	2.13
Cj1354	*ceuD*	enterochelin uptake ATP-binding protein	11.92
Cj1364c	*fumC*	fumarate hydratase	2.27
Cj1373		putative integral membrane protein	6.29
Cj1383c		hypothetical protein	132.52
Cj1384c		hypothetical protein	143.09

Cj1502c	*putP*	putative sodium/proline symporter	5.52
Cj1503c	*putA*	putative proline dehydrogenase/delta-1-pyrroline-5-carboxylate dehydrogenase	6.17
Cj1537c	*acs*	acetyl-CoA synthetase	5.78
Cj1541		LamB/YcsF family protein	2.85
Cj1548c		putative NADP-dependent alcohol dehydrogenase	2.51
Cj1613c	*chuZ*	putative pyridoxamine 5'-phosphate oxidase	9.71
Cj1614	*chuA*	haemin uptake system outer membrane receptor	127.69
Cj1615	*chuB*	putative haemin uptake system permease protein	33.45
Cj1617	*chuD*	putative haemin uptake system periplasmic haemin-binding protein	47.21
Cj1628	*exbB2*	putative exbB/tolQ family transport protein	56.49
Cj1629	*exbD2*	putative exbD/tolR family transport protein	29.81
Cj1651c	*map*	methionine aminopeptidase	6.07
Cj1659	*p19*	periplasmic protein p19	3.60
Cj1660		putative integral membrane protein	3.08
Cj1661		possible ABC transport system permease	3.54
Cj1662		putative integral membrane protein	3.50
Cj1663		putative ABC transport system ATP-binding protein	3.22
Cj1664		putative periplasmic thiredoxin	3.27

Table S5. Primer for qRT-PCR used in this study

Gene	FP	RP
FurR	GAGCTTGCCAATAAACCTCA	CAATCAAGGCTTGCTGTCTT
PerR	AAAAGCTACTCCGCAAAGACT	CCGTCGCTAAAGAGATTGAA
FeoB	CCAAGTGAAAAAGCAGGAAA	AAGGTTCATCAAGTCCACGA
CfrA	CCAGGCGTTGATTTATATGC	CCCATCAATCAAAACCAAAG
ChuA	AGCAAAAATACCAGCAGTGG	AAAGAAATTTCAGGGCGATT
p19	CAATCCAAATGGTTTTCCAG	ATTGCACCTGTGTCGGTATT
cfbpA	TAGCTTCGCCTGCAAATACT	GGCTAAAGGTGTGCTTGAAA
Sdac	GCAAAATAGCCAAAACAGGA	CAGCGATCATTACCTTTGCT
DcuB	GCATCCTTGGACTTATGCTG	CACCAATTCCTAAAGCCAAA
FrdB	AGATCCGCATGACCATAGAA	TCTTCACAAGCAAGCAATGA
oorA	GCGAGCTTAATATGGGACAA	GATAGGACGACCATTTGCAC
16SrRNA	TACCTGGGCTTGATATCCTA	GGACTTAACCCAACATCTCA

Table S6. Protein identification of spots of 2D-DIGE

Spot ID	IA3902 / NCTC11168	IA5908 / NCTC11168	IA3902	NCTC11168	Gene	Description	Theoretical MW (Da)	Theoretical pI	Comments
1	3.18	-1.56	N/A	N/A					Not identified due to inconsistency between abortion strains relative to NCTC11168
2	-9.41	-9.53		cj1729c	*flgE*	flagellar hook subunit protein	91918.97	4.86	78% identical to CJSA_1632, which is spot 3 in IA3902.
3	3.75	3.34	CJSA_1632		*flgE*	flagellar hook protein FlgE	90824.07	5.19	78% identical to Cj1729c; which is spot 2 in 11168
4	-11.39	-8.14	N/A	N/A					no confidence in protein ID
5	2.75	1.84	N/A	N/A					no confidence in protein ID
6	-2.83	-2.6	N/A???	cj 0129c		OMP85 outer membrane protein	83197	5.5	99% identical to CJSA_0120, which is spot 7 in IA3902
7	3.55	3.63	CJSA_0120			OMP85 family outer membrane protein	83127	5.6	99% identical to Cj0129c, which is spot 6 in 11168.
8	-9.25	-6.65	N/A	N/A					no confidence in protein ID
9	-10.25	-8	CJSA_0712	cj 0755	*cfrA*	ferric enterobactin uptake receptor CfrA	77554.48	5.29	94% identity; CJSA_0712, pI/mw: 5.41 / 77785.97
10	-1.59	2.2	N/A	N/A					Not identified due to inconsistency between abortion strains relative to NCTC11168
11	2.09	1.73	N/A	N/A					Not identified due to inconsistency between abortion strains relative to NCTC11168
12	-1.89	-1.95		cj 0518	*htpG*	heat shock protein 90 HtpG	69654.39	5.09	98% identical to CJSA_0488, which is spot 14 in IA3902
13	2.08	1.99	CJSA_0488		*htpG*	heat shock protein 90 HtpG	69600.35	5.12	98% identical to Cj0518 , which is spot 13 in 11168.
14	3.25	3.3	CJSA_0607		*aspS*	aspartyl-tRNA synthetase	66150.87	5.71	99% identical to Cj0640c , which is spot 16 in 11168.
15	-5.02	-6.78		cj 0640c	*aspS*	aspartyl-tRNA synthetase	66166.92	5.8	99% identical to Cj0640c , which is spot 15 in IA3902
16	-6.02	-4.3	CJSA_1526	cj 1614	*chuA*	haemin uptake system outer membrane receptor ChuA	80940.38	6.26	100% identity
17	-3.21	-4.79	N/A	N/A					no confidence in protein ID
18	-1.5	-2.05	N/A	N/A					no confidence in protein ID
19	-5.17	-8.86		cj 1339c	*flaA*	flagellin flaA	59038.66	5.59	93% identical to CJSA_1274, which is spot 21 in IA3902.
20	2.89	1.77	CJSA_1274		*flaA*	flagellin FlaA	59753.54	6.52	93% identical to Cj1339c, which is spot 20 in 11168.
21	2.45	2.52	CJSA_1274		*flaA*	flagellin FlaA	59753.54	6.52	93% identical to Cj1339c, which is spot 20 in 11168.
22	3.03	-1.52	N/A	N/A					Not identified due to inconsistency between abortion strains relative to NCTC11168
23	2.31	2.54	N/A	N/A					no confidence in protein ID
24	2.92	2.68	CJSA_0652		*pta*	phosphate acetyltransferase Pta	56178.69	5.54	99% identical to Cj0688 , which is spot 30 in 11168.
25	2.27	2.31	CJSA_0652		*pta*	phosphate acetyltransferase Pta	56178.69	5.54	99% identical to Cj0688 , which is spot 30 in 11168.
26	-2.47	-2.28		cj 0688	*pta*	putative phosphate acetyltransferase Pta	56177.75	5.75	99% identical to CJSA_0652 , which is spot 28, 29 in IA3902.
27	-2.2	-2.3		cj 1058c	*guaB*	inosine-5'-monophosphate dehydrogenase GuaB	52151.21	7.15	99 % identical to CJSA_1001, which is spot 32 in IA3902
28	3.42	3	CJSA_1001		*guaB*	inosine-5'-monophosphate dehydrogenase GuaB	52150.22	7.66	99 % identical to Cj1058, which is spot 31 in 11168.
29	-1.76	-1.86		cj 0662c	*hslU*	ATP-dependent Hsl protease ATP-binding subunit	49779.51	5.71	99% identical to CJSA_0627 , which is spot spot 34 in IA3902.
30	3.84	3.27	CJSA_0627		*hslU*	ATP-dependent protease ATP-binding subunit HslU	49778.52	5.84	99% identical to Cj0662c , which is spot 33 in 11168.
31	-3.98	-4.51		cj 1170c	*omp50*	50 kda outer membrane protein precursor Omp50	53339.85	6.14	88% identical to CJSA_1108 , which is spot 36 in IA3902.
32	3.96	4.04	CJSA_1108		*omp50*	50 kda outer membrane protein precursor Omp50	53123.68	6.28	88% identical to Cj1170 , which is spot 35 in 11168.
33	-2.59	-1.9		cj 1259	*porA*	Major outer membrane protein Momp	45687.52	4.71	95% identical to CJSA_1198,which is spot spots 39/40 in IA3902.
34	-43.42	-11.09		cj 1259	*porA*	Major outer membrane protein Momp	45687.52	4.71	95% identical to CJSA_1198,which is spot spots 39/40 in IA3902.
35	4.93	8.6	CJSA_1198		*porA*	Major outer membrane protein Momp	45706.61	4.87	95% identical to cj 1259,which is spot spots 37/38 in 11168.
36	37.45	4.82	CJSA_1198		*porA*	Major outer membrane protein Momp	45706.61	4.87	95% identical to cj 1259,which is spot spots 37/38 in 11168.
37	-34.99	-42.62	CJSA_0440	cj 0470	*tuf*	translation elongation factor Tu	43593.62	5.11	100% identity
38	4.37	3.89	CJSA_0596			prophage Lp2 protein 6	40822.58	5.09	Absent in 11168
39	-3.03	-3.79	CJSA_1119	cj 1181c	*tsf*	elongation factor Ts	39555	5.24	99% identity; CJSA_1119, pI/mw: 5.24 / 39524.98
40	-6.35	-5.15		cj 1110c		MCP-type signal transduction protein	48350.55	6	Pseudogene in IA3902 (CJSA_1052).
41	-23.29	-20.36		cj 1110c		MCP-type signal transduction protein	48350.55	6	Pseudogene in IA3902 (CJSA_1052).
42	4.12	3.73	CJSA_1232	Cj1294	*pseC*	C4 aminotransferase specific for PseB product (part of O-linked glycosylation)	42300.61	6.29	96% identity;Cj1294, pI/Mw: 6.05 / 42449.81
43	-2.71	-3.63		cj 1429c		hypothetical protein (CPS locus)	35757.83	5.25	Absent in IA3902.
44	5.94	6.1	CJSA_0966		*asd*	aspartate-semialdehyde dehydrogenase	38376.31	5.6	99% identical to Cj1023c , which is spot 49 in 11168
45	-10.71	-7.84		cj 1023c	*asd*	aspartate-semialdehyde dehydrogenase	38375.37	5.89	99% identical to CJSA_0966 , which is spot 48 in IA3902.
46	-4.13	-4.97	CJSA_0137	cj 0146c	*trxB*	thioredoxin reductase TrxB	33118.15	5.6	100% identity
47	-5.45	-5.15	CJSA_0137	cj 0146c	*trxB*	thioredoxin reductase TrxB	33118.15	5.6	100% identity
48	4.91	5.15	CJSA_0246		*ilvE*	branched-chain amino acid aminotransferase	33843.81	5.93	99% identical to cj 0269, which is spot 55 in 11168.
49	4.68	4.22	CJSA_0246		*ilvE*	branched-chain amino acid aminotransferase	33843.81	5.93	99% identical to cj 0269, which is spot 55 in 11168.
50	-3.67	-5.6	N/A	N/A					no confidence in protein ID
51	-7.54	-4.1		cj 0269	*ilvE*	branched-chain amino acid aminotransferase	33857.88	6.12	99% identical to CJSA_0246, which is spots 52/53 in IA3902
52	-5.02	-5.25	N/A	N/A					no confidence in protein ID
53	-4.07	-5.05	CJSA_0134	cj 0143c	*znuA*	putative periplasmic solute binding protein for ABC transport system	34577.72	6.71	98% identity; CJSA_0134, pI/Mw: 6.54 / 34625.71
54	-3.67	-4.79	CJSA_1159	cj 1221	*groEL*	60 kD chaperonin (cpn60) GroEL	57970.57	5.02	100% identity
55	-3.08	-2.64	N/A	N/A					no confidence in protein ID
56	-3.27	-3.66	CJSA_1159	cj 1221	*groEL*	60 kD chaperonin (cpn60) GroEL	57970.57	5.02	100% identity
57	4.23	4.61	CJSA_0810	Cj0855	*folD*	folD bifunctional protein (involved in folic acid biosynthesis)	30364.44	5.61	98% identity; Cj0855, pI/Mw: 5.90 / 30349.48
58	3.7	2.16	CJSA_1350	Cj1419c		Putative methyltransferase (part of CPS)	29704.62	5.92	100% identity
59	2.68	3.21	CJSA_0482		*purC*	phosphoribosylaminoimidazole-succinocarboxamide synthase PurC	26954.15	5.8	99% identical to Cj0512 , which is spot 66 in 11168.
60	-5.52	-5.67		cj 0512	*purC*	phosphoribosylaminoimidazole-succinocarboxamide synthase PurC	26938.19	5.8	99% identical to CJSA_0482 , which isspot 65 in IA3902.
61	5.47	3.11	CJSA_1350	Cj1419c		Putative methyltransferase (part of CPS)	29704.62	5.92	100% identity
62	-5.19	-3.91	CJSA_1525	cj 1613c		putative pyridoxamine 5'-phosphate oxidase	28670.53	6.16	100% identity
63	-3.04	-3.16	CJSA_1351	cj 1420c		putative methyltransferase (part of CPS)-	29570.7	6.26	99% identity; CJSA_1351, pI/Mw: 6.26 / 29558.65
64	7.45	7.95	CJSA_0329	Cj0355c		DNA-binding response regulator	25573.26	5.09	99% identity; Cj0355c, pI/Mw: 5.28 / 25542.29
65	31.56	37.16	CJSA_0329	Cj0355c		DNA-binding response regulator	25573.26	5.09	99% identity; Cj0355c, pI/Mw: 5.28 / 25542.29
66	8.66	6.78	CJSA_0329	Cj0355c		DNA-binding response regulator	25573.26	5.09	99% identity; Cj0355c, pI/Mw: 5.28 / 25542.29
67	-21.98	-27.65	CJSA0329	cj 0355c		DNA-binding response regulator	25573.26	5.09	99% identity; Cj0355c, pI/Mw: 5.28 / 25542.29

68	2.42	-2.18	N/A	N/A					Not identified due to inconsistency between abortion strains relative to NCTC11168
69	2.16	-3.23	N/A	N/A					Not identified due to inconsistency between abortion strains relative to NCTC11168
70	3.22	4.27	CJSA_0505	Cj0538	*oorC*	2-oxoglutarate-acceptor oxidoreductase subunit OorC	20123.16	5.5	99% identity; Cj0538, pI/Mw:5.68 / 20097.13
71	-11.96	-14.41	CJSA_0308	cj 0334	*aphC*	alkyl hydroperoxide reductase AphC	21947.14	5.66	99% identity; CJSA_0308, pI/Mw: 5.95 / 22046.27
72	6.87	9.4	CJSA_0505	Cj0538	*oorC*	2-oxoglutarate-acceptor oxidoreductase subunit OorC	20123.16	5.5	99% identity; Cj0538, pI/Mw:5.68 / 20097.13
73	-2.6	-2.25	CJSA_0308	cj 0334	*aphC*	alkyl hydroperoxide reductase AphC	21947.14	5.66	99% identity; CJSA_0308, pI/Mw: 5.95 / 22046.27
74	-14.49	-15.74	CJSA_0308	cj 0334	*aphC*	alkyl hydroperoxide reductase AphC	21947.14	5.66	99% identity; CJSA_0308, pI/Mw: 5.95 / 22046.27
75	-4.1	-6.06	CJSA_0505	cj 0538	*oorC*	2-oxoglutarate-acceptor oxidoreductase subunit OorC	20097.13	5.68	99% identity; CJSA_0505, pI/Mw:5.5/20123.16
76	10.12	9.86	CJSA_0308	Cj0334	*aphC*	alkyl hydroperoxide reductase AphC	22046.27	5.95	99 % identity; Cj0334, pI/Mw: 5.66/21947.14
77	6.43	8.28	CJSA_0308	Cj0334	*aphC*	alkyl hydroperoxide reductase AphC	22046.27	5.95	99 % identity; Cj0334, pI/Mw:5.66/21947.14
78	2.32	-4.17	N/A	N/A					Not identified due to inconsistency between abortion strains relative to NCTC11168
79	-2.8	-2.75	CJSA_1570	cj 1659	*p19*	periplasmic protein p19 (iron transport protein)	19642.49	5.23	100% identity
80	-4.77	-3.06	N/A	N/A					no confidence in protein ID
81	-12.72	-10.5	N/A	N/A					no confidence in protein ID
82	-3.44	-5.64	N/A	N/A					no confidence in protein ID
83	-2.87	-6.04	N/A	N/A					no confidence in protein ID

Note:N/A,no availabe data due to no confidence in protein ID or inconsistency between abortion strains relative to NCTC11168

Table S7. Differentially expressed proteins between IA3902 and NCTC11168 by 2D_DIGE

	Spots	NCTC11168	IA3902	Genes	Annotation	M_r	pI	Fold change (2D_DIGE)	Fold Change (Microarray)	Remarks
Increased in IA3902	spot38		CJSA0596		prophage Lp2 protein 6	40822.58	5.09	4.37	N/A*	IA3902 specific gene
	spot42	Cj1294	CJSA_1232	*pseC*	C4 aminotransferase specific for PseB product	42300.61	6.29	4.12	↑ 1.33*	97% probe identity
	spot57	Cj0855	CJSA_0810	*folD*	folD bifunctional protein	30364.44	5.61	4.23	↓ 2.14*	98% probe identity,
	spot58/61	Cj1419c	CJSA_1350		Putative methyltransferase	29704.62	5.92	3.7	↓ 3.53*	98% probe identity
Decreased in IA3902	spot9	Cj0755	CJSA_0712	*cfrA*	ferric enterobactin uptake receptor CfrA	77554.48	5.29	10.25	↓ 77.88	
	spot16	Cj1614	CJSA_1526	*chuA*	haemin uptake system outer membrane receptor ChuA	80940.38	6.26	6.02	↓ 127.69	
	spot37	Cj0470	CJSA_0440	*tuf*	translation elongation factor Tu	43593.62	5.11	34.99	↓ 1.67*	p=0.12,q=0.128
	spot39	Cj1181c	CJSA_1119	*tsf*	elongation factor Ts	39555	5.24	3.03	↓ 1.80 *	p=0.67,q=0.34
	spot40/41	Cj1110c	CJSA_1052		putative MCP-type signal transduction protein	48350.55	6	6.35	↓ 25.41	
	spot43	Cj1429c			hypothetical protein	35757.83	5.25	2.71	↓ 1.25*	p=0.06, q=0.088
	spot46/47	Cj0146c	CJSA_0137	*trxB*	thioredoxin reductase TrxB	33118.15	5.6	4.13	↓ 4.676	
	spot53	Cj0143c	CJSA_0134	*znuA*	putative periplasmic solute binding protein for ABC transport system	34577.72	6.71	4.07	↓ 3.46	
	spot54/56	Cj1221	CJSA_1159	*groEL*	60 kD chaperonin (cpn60) GroEL	57970.57	5.02	3.67	↓ 1.48*	p=0.218,q=0.176274
	spot62	Cj1613c	CJSA_1525	*chuZ*	putative pyridoxamine 5'-phosphate oxidase	28670.53	6.16	5.19	↓ 9.7	
	spot63	Cj1420c	CJSA_1351		putative methyltransferase	29570.7	6.26	3.04	↓ 1.49*	p=0.148,q=0.143
	spot79	Cj1659	CJSA_1570	*p19*	periplasmic protein p19 (iron transport protein)	19642.49	5.23	2.8	↓ 3.60	

* Not reliable fold change due to the mismatch of probes to IA3902 or no cutoff (fold change > 2, *q* value < 0.1) qualified value in the microarray data
